# Changes in physiology and microbial diversity in larval ornate chorus frogs are associated with habitat quality

**DOI:** 10.1093/conphys/coaa047

**Published:** 2020-06-15

**Authors:** Cory B Goff, Susan C Walls, David Rodriguez, Caitlin R Gabor

**Affiliations:** 1 Department of Biology, Texas State University, 601 University Dr. San Marcos, TX 78666, USA; 2 Department of Biology and Chemistry, Liberty University, 1971 University Blvd. Lynchburg, VA 24515, USA; 3 Wetland and Aquatic Research Center, U.S. Geological Survey, 7920 NW 71st St. Gainesville, FL 32653, USA

**Keywords:** Bacterial communities, corticosterone, environmental stress, water-borne hormones

## Abstract

Environmental change associated with anthropogenic disturbance can lower habitat quality, especially for sensitive species such as many amphibians. Variation in environmental quality may affect an organism’s physiological health and, ultimately, survival and fitness. Using multiple health measures can aid in identifying populations at increased risk of declines. Our objective was to measure environmental variables at multiple spatial scales and their effect on three indicators of health in ornate chorus frog (*Pseudacris ornata*) tadpoles to identify potential correlates of population declines. To accomplish this, we measured a glucocorticoid hormone (corticosterone; CORT) profile associated with the stress response, as well as the skin mucosal immune function (combined function of skin secretions and skin bacterial community) and bacterial communities of tadpoles from multiple ponds. We found that water quality characteristics associated with environmental variation, including higher water temperature, conductivity and total dissolved solids, as well as percent developed land nearby, were associated with elevated CORT release rates. However, mucosal immune function, although highly variable, was not significantly associated with water quality or environmental factors. Finally, we examined skin bacterial diversity as it aids in immunity and is affected by environmental variation. We found that skin bacterial diversity differed between ponds and was affected by land cover type, canopy cover and pond proximity. Our results indicate that both local water quality and land cover characteristics are important determinants of population health for ornate chorus frogs. Moreover, using these proactive measures of health over time may aid in early identification of at-risk populations that could prevent further declines and aid in management decisions.

## Teaser

We examined multiple health measures in ornate chorus frog tadpoles collected from across their range as a function of water quality and land cover characteristics. Water quality, land cover, developed land and location were linked to health indicators, suggesting these measures may aid in identifying populations at increased decline risk.

## Introduction

As the human population continues to increase, land-use conversion has altered habitat suitability for many species (Vitousek *et al.*, 1997; McKinney, 2002). Habitat loss, fragmentation and degradation, coupled with climate change and other anthropogenic factors, are among the most significant drivers of population declines and species extinctions (Brook *et al.*, 2008; Mantyka-Pringle *et al.*, 2012; Segan *et al.*, 2016; Ceballos *et al.*, 2017). Landscape-level disturbances such as habitat loss and degradation affect populations by altering habitat connectivity, composition and quality (Fahrig, 2003). For aquatic organisms, local impacts on water quality such as changes in temperature, pH, contaminant, nutrient and sediment levels (Meybeck, 2004) can also pose significant consequences to population health and resilience. Together, the additive or synergistic effects are especially troubling for species, especially those with complex life cycles that require multiple habitats to complete development (Hayes *et al.*, 2010; Blaustein *et al.*, 2011). Examining physiological responses of individuals to such factors can provide insight into the mechanisms by which environmental stressors can lead to population declines (Wikelski and Cooke, 2006). In turn, understanding the consequences of these factors on populations could aid in better management practices for declining species.

Glucocorticoid (GC) hormones can be useful tools to assess the physiological response of organisms to environmental and anthropogenic stressors which, when combined with other metrics (e.g., immune function), can be indicators of individual and population health (Sheriff *et al.*, 2011; Dantzer *et al.*, 2014; Jeffrey *et al.*, 2015). In response to stressors, GC hormones are released by the hypothalamo-pituitary-interrenal (HPI) axis above baseline levels as an adaptive response that assists in energy mobilization, mediates natural changes in physiology and behaviour, kick-starts the immune response and helps return the organism to homeostasis (Dhabhar and McEwen, 1999; Sapolsky *et al.*, 2000; Cyr and Romero, 2009; Romero *et al.*, 2009). However, under prolonged (chronic) stress, vertebrates may have elevated or lowered baseline GCs. In such cases, the HPI axis can become dysregulated (where individuals have a muted response to additional stressors), resulting in reduced physiological health, pathology and ultimately death (Wingfield and Romero, 2001; McEwen and Wingfield, 2003; Cyr and Romero, 2009; Romero *et al.*, 2009). Vertebrates under chronic stress have suppressed growth and development, reduced reproduction and reduced immune function (Sapolsky *et al.*, 2000; McEwen and Wingfield, 2003; Romero, 2004; Rollins-Smith, 2017). Pre-stressor baseline GC levels and the response to acute stressors (HPI axis responsiveness) can be measured to aid in identifying whether populations are under chronic stress, which may be associated with higher risk of declines (Homan *et al.*, 2003; Dantzer *et al.*, 2014; Baugh *et al.*, 2018; Gabor *et al.*, 2018). Measuring GC hormones may identify populations to target for management but may not provide a full picture of environmental effects on population health. Using additional metrics such as immune function and microbial diversity is needed for a more holistic view of how environmental stressors impact population health (Breuner *et al.*, 2013).

Amphibians have the highest threat status of all vertebrate classes (Stuart *et al.*, 2004; Foden *et al.*, 2013) and are particularly susceptible to changes in habitat quality. Species that are especially vulnerable use ponds or other water bodies to breed but occupy terrestrial habitats outside the breeding season (Becker *et al.*, 2007). As such, the quality and conditions of both the aquatic and surrounding terrestrial environments play a factor in the health of these amphibians. Environmental changes and conditions associated with lower quality habitat may be perceived as stressors, which elevate or dysregulate GC hormones and affect physiological health (Homan *et al.*, 2003; Janin *et al.*, 2011, 2012; Chambers *et al.*, 2013; Gabor *et al.*, 2018). Amphibians release the hormone corticosterone (CORT; the primary amphibian GC) when exposed to various stressors, including increased pond drying, high salinity, extreme pH and temperature and other water quality variables (Denver, 1998; Chambers *et al.*, 2013; Gomez-Mestre *et al.*, 2013; Narayan and Hero, 2014a, b; Burraco and Gomez-Mestre, 2016; Gabor *et al.*, 2018). In addition, landscape characteristics such as the extent of canopy cover and forest fragmentation around breeding ponds, as well as substrate type, are significant predictors of CORT in adult common toads (*Bufo bufo*; Janin *et al.*, 2011, 2012) and spotted salamanders (*Ambystoma maculatum*; Homan *et al.*, 2003), and increased CORT in Jollyville Plateau salamanders (*Eurycea tonkawae*) has been associated with more urbanized streams (Gabor *et al.*, 2018). Landscape-level factors may also influence pond quality, thus affecting aquatic larvae indirectly. Acute changes in pond water salinity can be lethal for larval amphibians (Brown and Walls, 2013). Canopy cover is known to influence amphibian assemblages, with higher amphibian diversity and faster growth in open-canopy wetlands (Werner and Glennemeier, 1999; Skelly *et al.*, 2005). However, increased temperatures associated with climate change combined with open canopy wetlands may reduce wetland hydroperiod, which negatively affects juvenile recruitment and larval survival (Semlitsch *et al.* 1996; Daszak *et al.* 2005). These findings suggest a link between habitat quality at multiple spatial scales and physiological health in amphibians.

Microbiota play a role in amphibian immune response to disease (Harris *et al.*, 2009; Woodhams *et al.*, 2016) and may vary with abiotic factors (Bletz *et al.*, 2017a; Jani and Briggs, 2018; Varela *et al.*, 2018), across populations (Kueneman *et al.*, 2013; Hernandez-Gomez *et al.*, 2017; Jani and Briggs, 2018) and among species (Kueneman *et al.*, 2013; Belden *et al.*, 2015; Varela *et al.*, 2018). Microbiota living on the skin combined with skin secretions make up the mucosome, or micro-ecosystem of the skin, providing the first line of defence in amphibian disease resistance (Woodhams *et al.*, 2007; Harris *et al.*, 2009; Woodhams *et al.*, 2014). Identifying the bacterial community of amphibians in a population and the ability of their mucosome to fight infection (mucosome function; Woodhams *et al.*, 2014) can be used to predict disease risk across populations (Woodhams *et al.*, 2014). Elevated CORT over prolonged periods is associated with disease (Warne *et al.*, 2011; Gabor *et al.*, 2013b; Peterson *et al.*, 2013; Gabor *et al.*, 2015, 2018; Kindermann *et al.*, 2017), is immunosuppressive (reviewed in Rollins-Smith, 2017), inhibits production of amphibian skin peptides (Rollins-Smith *et al.*, 2011) and affects gut microbial communities in other taxa (Clarke *et al.*, 2014). Further, the microbiome of amphibians shifts with temperature (Kohl and Yahn, 2016), soil pH, precipitation (Varela *et al.*, 2018) and infection intensity (Jani and Briggs, 2018). Therefore, reduced habitat quality and increased stress from environmental stressors may be associated with altered skin bacterial communities and immunity in amphibians, which could lead to increased declines.

To explore potential underlying causes of population declines in the ornate chorus frog (*Pseudacris ornata*), we measured environmental variables at multiple spatial scales, including pond water quality and surrounding landscape characteristics, and examined their effect on three health metrics in tadpoles. Because of the known interactions between habitat quality and population health, we measured and examined the relationships of these variables with CORT release rates, mucosome function, and bacterial community diversity from populations of *P. ornata* across their range. We tested several hypotheses to assess whether environmental quality affects tadpole health: first, we quantified baseline and acute stress-induced CORT release rates for tadpoles collected across the species’ range as a measure of the CORT profiles for a pond. We hypothesized that habitat quality at multiple spatial scales affects CORT release rates and therefore the CORT profile in *P. ornata* larvae, as environmental conditions are associated with altered physiological health in amphibians (Homan *et al.*, 2003; Janin *et al.*, 2011, 2012; Chambers *et al.*, 2013; Gabor *et al.*, 2018). We measured pond water quality [temperature, pH, conductivity and total dissolved solids (TDSs)] as well as land cover characteristics (percent canopy, land cover type and percent developed land) at three spatial scales surrounding each pond. We also tested the hypotheses that these environmental conditions and CORT release rates are associated with altered immune function and an altered skin bacterial community, as both environment and stress are associated with altered immune defences (Rollins-Smith, 2017; Bletz *et al.*, 2017a; Jani and Briggs, 2018; Varela *et al.*, 2018). Our examination of multiple metrics of population health allows us to potentially identify links between environmental stress from reduced habitat quality and population declines which could aid in management practices.

## Materials and methods

### Study species

The ornate chorus frog (*P. ornata*) is endemic to the southeastern Coastal Plain and longleaf pine (*Pinus palustris*) ecosystem of the southeastern USA (Means, 2006). This frog has a disjunct range that extends from North Carolina south to Florida and west to Louisiana (Enge *et al.*, 2014; Powell *et al.*, 2016). The ornate chorus frog is a longleaf pine specialist common throughout the panhandle and northern regions of Florida in mesic and xeric habitats, but this species has been declining along its southern range edge in the Florida peninsula and western edge in Louisiana (Bartlett and Bartlett, 2011; Enge *et al.*, 2014). Declines are potentially caused by increased temperatures, droughts and reduced habitat quality associated with fire suppression (Means and Simberloff, 1987; Enge *et al.*, 2014). Fire suppression and insufficient management can reduce environmental quality for species endemic to pyrogenic systems, such as *P. ornata* and the longleaf pine-wiregrass (*P. palustris-Aristida* spp.) ecosystem of the southeastern USA (Noss, 2013, 2018). Hydroperiod is also considered an important factor associated with *P. ornata* population persistence, as this species requires fishless, seasonally inundated water bodies with a 3- to 4-month hydroperiod for complete tadpole development (Semlitsch *et al.*, 1996; Enge *et al.*, 2014). Though little is known about the adult frog, it is known to be fossorial, using loose, sandy soils to burrow and has been found over 400 m from breeding ponds (Brown and Means, 1984). Adults are winter breeders, with males calling as early as November, with actual breeding occurring December or January through March, when females deposit eggs on submerged vertical vegetation (Dorcas and Gibbons, 2008; Enge *et al.*, 2014). Roadside ditches, flooded fields and marshes adjacent to forested areas, as well as pine and mixed-woodland ponds can serve as aquatic breeding sites (Dorcas and Gibbons, 2008; Bartlett and Bartlett, 2011; Enge *et al.*, 2014; Powell *et al.*, 2016). However, most breeding ponds are found within sandhills, flatwoods, wetlands and pine forest/plantations and are associated with open-canopy sites with herbaceous understory, characteristic of a short-interval fire regime (Gorman *et al.*, 2013; Noss, 2013; Enge *et al.*, 2014; Noss, 2018).

### Field sampling

**Figure 1:**
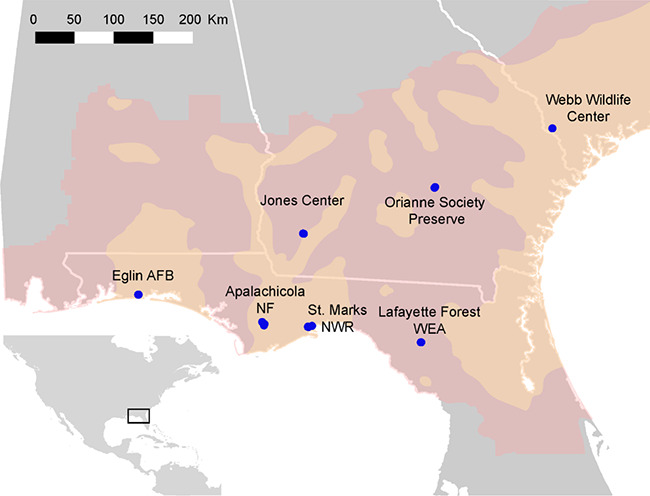
Approximate pond locations (blue dots) throughout the range of *Pseudacris ornata*. Pink shading represents historical range (International Union for the Conservation of Nature [IUCN], 2019) and orange shading represents newest range map (Powell *et al.*, 2016). Ponds are located on the following properties: Apalachicola National Forest (NF), Eglin Air Force Base (AFB), Joseph W. Jones Ecological Research Center at Ichauway, Lafayette Forest Wildlife Environmental Area (WEA), St. Marks National Wildlife Refuge (NWR), Orianne Society Preserve and the James W. Webb Wildlife Center.

We collected tadpoles (stages 30–40; Gosner, 1960) across 7 properties and up to 4 ponds (Sites) per property (Property) for a total of 16 ponds throughout the species’ range in Florida, Georgia and South Carolina (Table 1; Fig. 1). Ponds were sampled 2–23 March 2016 and 23 February–14 March 2017 (Table 1). At each pond, we recorded water temperature, conductivity and TDS; pH was also collected from ponds in 2017 (Table 2). Additionally, we conducted a geographic information systems analysis of land cover at each pond using land cover class and percent canopy cover data from the National Land Cover Database (NLCD, 2011). Using ArcMap (ESRI, Redlands, CA), we created a 100, 500 and 1000 m buffer around each pond. Land cover classes were determined for each 30 m pixel within the three spatial scales. The 16 land cover types recognized by the NLCD were combined into six classes for analysis: Agriculture, Developed, Forest, Shrub, Water and Wetlands. The Agriculture class included grasslands, pasture and cultivated crops; Developed included all intensities of developed and barren land; Forest included deciduous, evergreen and mixed forest cover; Shrub consisted of short, woody plants; Water was characterized by open ponds; and Wetlands included woody and emergent herbaceous wetlands. We then quantified the percent of each land cover type and the percent canopy cover within the three spatial scales using the zonal statistics as a table tool in ArcMap. We used the primary land cover class, percent developed land and percent canopy cover for each of the three spatial scales in analyses.

**Table 1:** Pond property, state, sites within each property and dates sampled

Region	State	Site	Dates sampled
Eglin Air Force Base	FL	EG1	2 March 2016 and 23 February 2017
		EG2	24 February 2017
Apalachicola National Forest	FL	AP1	3 March 2016 and 26 February 2017
		AP2	4 March 2016 and 25 February 2017
		AP3	4 March 2016
St. Marks National Wildlife Refuge	FL	SM1	8 March 2016
		SM2	8 March 2016
		SM3	28 February 2017
		SM4	1 March 2017
Joseph W. Jones Ecological Research Center at Ichauway	GA	JC1	22 March 2016 and 4 March 2017
		JC2	22 March 2016 and 2 March 2017
		JC3	23 March 2016 and 3 March 2017
Lafayette Forest Wildlife Environmental Area	FL	LF1	15–16 March 2016
Orianne Society Preserve	GA	OS1	9 March 2017
		OS2	10 March 2017
James W. Webb Wildlife Center	SC	WC1	14 March 2017

**Table 2:** Water quality variable ranges and average values across all sites sampled for each year

Variable	Range		Average	
	2016	2017	2016	2017
Water Temperature (°C)	13.6–23.1	11.3–24.3	17.2	19.2
Conductivity (μs/cm)	12.1–49.4	13.0–48.7	22.8	25.5
pH	N/A	4.12–5.95	N/A	5.17

### Water-borne CORT release rates

In 2016 and 2017, we used a non-invasive water-borne hormone method (Gabor *et al.*, 2013a, 2016) to measure CORT release rates from up to 40 individual tadpoles from each pond (Supplementary Table S1). This method measures CORT secreted through the skin, urine and faeces, which provides an integrated measure of the cumulative effects of chronic stress (Sheriff *et al.*, 2011; Dantzer *et al.*, 2014). We captured tadpoles using dipnets and then placed individuals in 250 ml beakers, one individual per beaker, containing 100 ml of bottled spring water and a perforated Nalgene liner. This liner allowed us to remove the tadpole but leave the water sample in the beaker. Half of the individuals were unmanipulated for 1 hour to obtain baseline CORT release rates and the other half were agitated by shaking the beakers for 1 min every 3 min for 1 hour to measure the response to this acute agitation stressor (Gabor *et al.*, 2016; Forsburg *et al.*, 2019). These baseline and agitation CORT release rates represent the treatment categories. After 1 hour, we removed the liner with the individual from each beaker and poured the water containing leached hormones into HDPE plastic cups. Samples were maintained on ice and transported to the laboratory for immediate extraction or frozen at −20°C to be processed at a later time (Ellis *et al.*, 2004). Frozen samples were extracted within 1 week of collection. We photographed each tadpole from the side with a ruler for scale before releasing it back into the pond. Snout-vent length (SVL, in mm) was measured from photographs using the program ImageJ (Rosband, 1997). In 2017, all tadpoles from which water-borne hormones were collected were also swabbed for skin bacterial community analysis. After the water samples containing leached hormones were collected, each individual was swabbed ventrally from the mouth to the vent using a single, sterile swab. The cotton tips of each swab were placed in Eppendorf tubes, frozen and transported back to the lab for analysis.

### Hormone extraction and validation

Once defrosted, we filtered each water sample through standard coffee filters (equivalent to grade 4 filter paper) to remove large debris and faecal material. All samples were then drawn through C18 solid phase extraction (SPE) columns (SepPak Vac 3 cc/500 mg; Waters, Inc.) primed with 4 ml methanol and 4 ml distilled water (Gabor *et al.*, 2016). After hormone extraction, SPE columns containing the hormone residue were stored at −20°C for up to 3 months before elution. We eluted columns with 4 ml methanol into borosilicate test tubes. Eluted samples were dried using nitrogen gas flowing through an Evap-O-Rac (Cole-Palmer) while sitting in a hot water bath (37°C). Each dried sample was then resuspended in a mixture of 5% ethanol and 95% enzyme-immunoassay (EIA) buffer (Cayman Chemical Company, Inc.) to a final volume of 260 μl. We also ran blank controls of bottled spring water resuspended to a final volume of 130 μl. We reconstituted at the minimum volume required to plate each sample in duplicate (with some leftover) because water samples have such low CORT values. The samples were resuspended to 260 μl to dilute the CORT concentrations to within the range of the standard curve. Each sample was run in duplicate on CORT EIA plates (*N* = 26; # 501320, Cayman Chemical Company, Inc.) and absorbance was read using a spectrophotometer plate reader (BioTek ELX 800) set to 405 nm. Final CORT values (pg/ml) were multiplied by amount resuspended (0.260 ml) and divided by SVL for final unit of pg/mm per hour. Water samples were multiplied by reconstitution volume (0.130 ml) and the relevant spring water values (5.4–15.6 pg/sample) were subtracted from the CORT release rates of each tadpole (adjusted range: 17.8–1957.4 pg/mm per hour).

The water-borne CORT collection method was validated for *P. ornata* using a pooled sample of hormones from seven non-experimental tadpoles serially diluted (following Gabor *et al.*, 2016). We examined parallelism of the serial dilution curve (1:1–1:32) of the pooled sample to the known standard curve (comparison of slopes, *t*_11_ = 1.304, *P* = 0.22). We also assessed quantitative recovery by spiking the pooled sample with each of eight standards. The observed recovery ranged from 62.0% to 82.3%, and we found a linear relationship between observed and expected slopes (β = 0.77, *F*_1,6_ = 1817.75, *R*^2^ = 0.997, *P* < 0.0001). Using a different pooled control sample run in quadruplicate on each plate, we examined the intra- and inter-plate variation of the 26 plates. Intra-plate variation ranged from 0.29–16.33% and mean inter-plate variation was 13.11%. The sensitivity of the CORT EIA plates ranged from 41.47 to 1390.60 pg/ml on average.

### Mucosome function

In 2017, 1 ml water was removed from each baseline CORT sample and stored at −20°C to assess mucosome function. A subset of these samples was used to assess mucosome function of individuals from each pond (Supplementary Table S1). Using the Cell Titer Glo 2 kit (Promega), a 25-μl water sample—containing host skin peptides, mucosal antibodies and bacterial communities—was combined with a 25-μl solution of *Batrachochytrium dendrobatidis* (*Bd*) zoospores with known concentration (approximately 25 000 total zoospores per 25 μl) on 96-well plates (Barnhardt, 2018). *Bd* zoospores were collected from plate cultures of isolate JEL423, which is within the Global Panzootic Lineage (BdGPL), by flooding 3- to 5-day-old culture plates with autoclaved water to stimulate zoospore release from zoosporangia. This *Bd* strain was used because it was readily available, easy to grow in the laboratory and detected across North America (Farrer *et al.*, 2011; Rosenblum *et al.*, 2013; Marshall *et al.*, 2019). Each mucosome sample was then plated in triplicate to assess *Bd* viability in the presence of the mucosome. In addition, six heat-killed *Bd* standards (0%, 20%, 40%, 60%, 80%, 100%) were plated in triplicate. After incubation for 1 hour at room temperature, 50 μl of Cell Titer Glo reagent was added to each well and placed on an orbital shaker at 200 rpm for 2 min and incubated at room temperature for an additional 15 min. A luminescent plate reader was then used (Biotek Synergy H1) to assess the percent cell viability from a ratio of live:dead *Bd* to determine the mucosome function against the pathogen. The gain of the plate reader was set at 150.

### Skin bacterial diversity

We extracted DNA from skin swabs of approximately 10 tadpoles per pond (Supplementary Table S1) using 50 μl of PrepMan (Applied Biosystems, Inc.) following the manufacturer’s protocol. Swabs and extracts were spun down briefly then the swab was inverted with sterile forceps and spun down again at 2 348 x g for 1 min. The swab was removed from the tube with sterile forceps and the remaining extract was centrifuged at 21 130 x g for 5 mins to pellet any precipitates. Without disturbing the pellet, 40 μl of extract was transferred to a new 1.5-ml centrifuge tube (adapted from Becker *et al.*, 2015). The purified extracts were used to generate PCR amplicons of bacterial 16S V4 properties using 515F and GOLAY barcoded 806R primers (Caporaso *et al.*, 2011) following the Earth Microbiome Protocol (Gilbert *et al.*, 2014). Amplicons were quantified using a Qubit dsDNA Broad sensitivity Assay Kit (Invitrogen) and, after normalizing and pooling, amplicons were size selected on a BluePippen (SageScience, Inc.) using a 2% agarose 100–600 Bp cartridge, the resulting fraction was cleaned using AMPure XP magnetic beads (Beckman Coulter, Inc). The pooled library size (~350 Bp) and concentration were verified on a TapeStation 2200 (Agilent Technologies, Inc.) using a D1000 screen tape and reagents. Sequencing was performed on a MiSeq instrument (Illumina, Inc.) using the 600-cycle MiSeq Reagent Kit v3. The resulting reads from the MiSeq were trimmed of adapters and parsed in BaseSpace (Illumina, Inc.) according to their respective barcode. The dada2 version 1.5.0 pipeline (Callahan *et al.*, 2016) in R (version 3.5.2; R Core Team, 2018) was used to first inspect read quality profiles and then filter and trim reads. Reverse reads were not used in downstream analyses owing to low quality, and thus, paired read merging was skipped after dereplication. Forward sequence reads were trimmed to 220 Bp based on their quality profile. Taxonomy assignment was performed using dada2 formatted FASTA file from the Silva taxonomic training data (version 132). The resulting dada2 tables were merged with the sample metadata and analysed using phyloseq (McMurdie and Holmes, 2013). Using phyloseq, samples were rarified to 25 000 sequences (average number of sequences = 229 663) and alpha diversity indices (Richness, Shannon and Simpson) were calculated. Richness is the number of species present, whereas Shannon diversity and Simpson diversity are based on the proportion of individuals of a particular species within the sample. The Shannon diversity index provides a value of diversity from estimating how likely an unknown individual is from a known species. Simpson diversity places a higher importance on dominant or more abundant species and is the probability two individuals are from different species. In these indices, a higher value (uncertainty/probability) indicates a higher diversity. Measuring all three indices provides a more complete picture of the bacterial diversity and composition on the amphibian skin. The final data set consisted of 8 337 operational taxonomic units (OTUs) across 98 samples.

### Statistical analysis

All analyses were run using R (version 3.5.2; R Core Team 2018). First, to determine if there was spatial autocorrelation in CORT profiles across ponds, distance-based Moran’s eigenvector mapping (MEM) was used to map longitude and latitude for each pond and build spatial predictors using the following packages: adespatial (Dray *et al.*, 2018), geosphere (Hijmans, 2017) and vegan (Oksanen *et al.*, 2019). Permutation analysis of variance (ANOVA) on the distance-based Moran axes identified two of three spatial axes as significant predictors of CORT variation. These two spatial axes, MEM2 and MEM3, corresponded to scaled distance measures of longitude and latitude, respectively. These axes were extracted and incorporated into model building. Eigenvalues for these two positive spatial axes were −0.78 and 0.24, respectively, indicating they represent autocorrelation between ponds at a fine spatial scale.

We developed linear mixed effect (lme) models in the package nlme (Pinheiro and Bates, 2018) to examine predictors of (i) CORT release rates, (ii) mucosome function and (iii) skin bacterial diversity indices (Richness, Shannon, Simpson) among ponds as separate response variables. All models for all response variables included Site as a random effect. CORT models examined natural log-transformed CORT release rates standardized by SVL (pg/mm per hour) as the response variable. Prior to building models, we first ran a preliminary analysis of models containing each land cover characteristic calculated from NLCD variables (landcover type, percent urban development and percent canopy cover) at each spatial scale as sole predictors for each of the response variables. To determine the most important scale for the three predictors, the models containing the landcover characteristic at the different scales were ranked according to Akaike’s information criterion adjusted for small sample size (AICc) and the scale included in the highest ranked model was retained for subsequent models. Water quality variables (water temperature, conductivity, TDS, pH) from all ponds were combined using a principle components analysis (PCA) to reduce the number of variables in analyses. We ran analyses to assess predictors of CORT release rates for each year (2016 and 2017) separately, as not all ponds were sampled in both years and pH was an additional pond characteristic added in 2017. For 2016, PCA revealed that PC1 accounted for 83.3% of the variation in the data and was driven by conductivity and TDS, whereas PC2 accounted for 16.7% of the variation and was mainly driven by water temperature (Table 3). In 2017, PC1 accounted for 59.6% of the variation and was driven by conductivity and TDS, whereas PC2 accounted for 26.4% of the variation and was driven by water temperature (Table 3). To test the relationship of predictors to each of the three response variables, models incorporated main effects including the dominant land cover type within 100 m of ponds (Landcover100), the percent developed land within 1000 m of ponds (Urban1000), the percent canopy cover within 500 m of ponds (Canopy500), PC1 and PC2 for each year, spatial axes (MEM2 and MEM3), the property each pond was located within (Property), as well as additive models. We created a total of 28 models for analyses (Supplementary Table S2). Additionally, we analysed each treatment (Baseline or Agitation) separately within each year. A similar list of models was used to examine predictors of mucosome function and skin microbial diversity (Supplementary Tables S3 and S4), substituting the correct scale of the landscape predictors as determined by separate preliminary analyses and adding baseline CORT release rates (BCORT) as a sole predictor in an additional model. Because other analyses were run to assess impacts of both Site and Property on skin bacterial diversity indices (PERMANOVA; see below), Property was not included as a predictor in the bacterial diversity models. This resulted in 29 models for mucosome analysis and 28 models for skin bacterial diversity analysis. We used each bacterial diversity index (Richness, Shannon and Simpson) as a separate response variable in separate analyses involving the entire model set. All models were ranked according to Akaike information criterion corrected for small sample size (AIC_c_) using the package MuMIn (Barton 2018). We calculated parameters using the maximum-likelihood estimation during the model-selection process. Model-averaged parameter estimates, unconditional standard error (SE) and unconditional 95% CIs were calculated for candidate models (ΔAICc < 2) using the package AICcmodavg (Mazerolle 2019). In addition, we ran independent *t*-tests to compare baseline and agitation-induced CORT release rates for each pond and year separately to specifically address which populations might be chronically stressed, indicated by an inability to mount a CORT response above baseline levels. We also ran an ANOVA and Levene’s test using the package lawstat (Gastwirth *et al.*, 2017) to examine differences in mean mucosome function and mean variance across Sites.

**Table 3:** Eigenvectors and principle components for PCA of water quality variables collected at ponds in 2016 and 2017

Variable	2016		2017	
	PC1	PC2	PC1	PC2
W_Temp	0.503	**−0.858**	−0.206	**0.876**
Conductivity	**0.620**	0.274	**−0.630**	0.116
TDS	**0.602**	0.435	**−0.614**	−0.091
pH			0.429	0.460

Variables that loaded heavily on components are in bold.

Means of each of the three alpha diversity indices were compared across Site and Property. We analysed mean Shannon diversity across Sites and Properties using an ANOVA. Because data were non-normal, we used a Kruskal–Wallis test to compare the mean Richness and Simpson diversity across Sites and Properties. The relative contribution of Site and Property to microbiome diversity (beta diversity) were analysed using PERMANOVA (Adonis; 999 permutations) using the package vegan (Oksanen *et al.*, 2019). All pairwise comparisons were assessed for significant factors using the package pairwiseAdonis (Martinez Arbizu, 2019). Beta diversity of skin bacterial communities (OTUs) were plotted using the Bray–Curtis method of non-metric multidimensional scaling (NMDS) and the following packages: phyloseq (McMurdie and Holmes, 2013) and ggplot2 (Wickham, 2016).

## Results

### Water-borne CORT release rates

**Figure 2:**
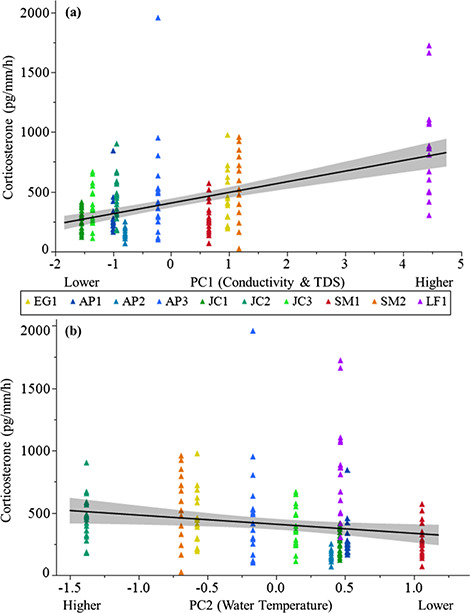
Relationship between agitation CORT release rates (pg/mm per hour) and (a) PC1 or (b) PC2 values. Data were collected from *P. ornata* tadpoles and ponds sampled in 2016. Untransformed data are shown. Ponds: AP = Apalachicola National Forest, EG = Eglin Air Force Base, JC = Joseph W. Jones Ecological Research Center at Ichauway, LF = Lafayette Forest Wildlife Environmental Area, SM = St. Marks National Wildlife Refuge.

**Figure 3:**
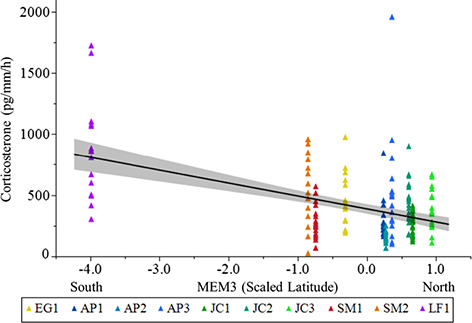
Relationship between agitation corticosterone release rates (pg/mm per hour) and MEM3 values. Data were collected from *P. ornata* tadpoles and ponds sampled in 2016. Untransformed data are shown. Ponds: AP = Apalachicola National Forest, EG = Eglin Air Force Base, JC = Joseph W. Jones Ecological Research Center at Ichauway, LF = Lafayette Forest Wildlife Environmental Area, SM = St. Marks National Wildlife Refuge.

**Figure 4:**
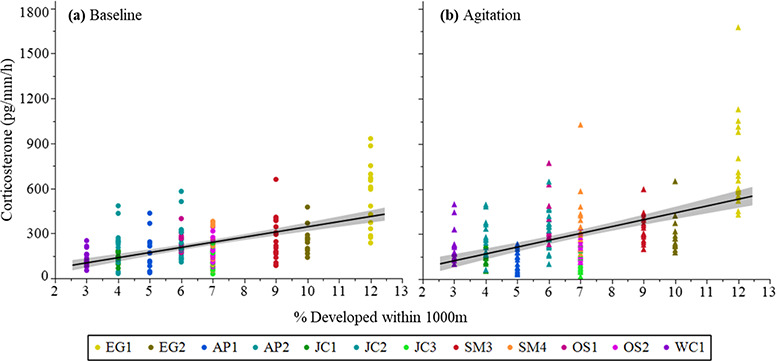
Relationship between (a) baseline and (b) agitation CORT release rates (pg/mm per hour) and the percent developed land within 1000 m. Data were collected from *P. ornata* tadpoles and ponds sampled in 2017. Untransformed data are shown. Ponds: AP = Apalachicola National Forest, EG = Eglin Air Force Base, JC = Joseph W. Jones Ecological Research Center at Ichauway, LF = Lafayette Forest Wildlife Environmental Area, SM = St. Marks National Wildlife Refuge.

**Figure 5:**
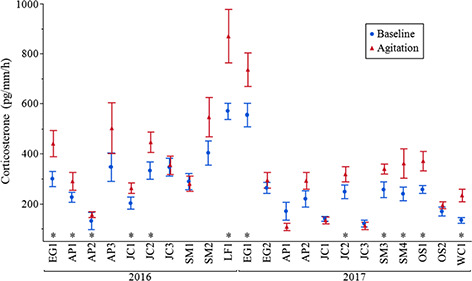
Mean (± SE) CORT release rates (pg/mm per hour; untransformed data shown) of *P. ornata* tadpoles for both treatments (Baseline or Agitation) at each pond across both years. Blue circles = Baseline values, Red triangles = Agitation values. Asterisks indicate significant differences. Ponds: AP = Apalachicola National Forest, EG = Eglin Air Force Base, JC = Joseph W. Jones Ecological Research Center at Ichauway, LF = Lafayette Forest Wildlife Environmental Area, SM = St. Marks National Wildlife Refuge, OS = Orianne Society Preserve, WC = James W. Webb Wildlife Center. Ponds are ordered geographically from west to east.

AICc model selection indicated six candidate models (ΔAICc < 2) to predict baseline CORT release rates and five candidate models to predict agitation CORT release rates for sites sampled in 2016 (Supplementary Table S5). Model averaged parameter estimates indicated both PCs and both spatial axes were top predictors of baseline CORT release rates, though no predictor was significant (CI did not overlap 0; Table 4). Model averaged parameter estimates indicated both PCs and MEM3 (scaled latitude) were significant predictors of agitation CORT release rates in 2016 (Table 4). Agitation CORT release rates were higher in ponds with higher conductivity and TDS (positive loading on PC1; Fig. 2a) and lower in ponds with lower water temperature (negative loading on PC2; Fig. 2b). Additionally, agitation CORT release rates were higher at lower MEM3 values (Fig. 3). For 2017, two models predicting baseline CORT release rates and two models predicting agitation CORT release rates had a ΔAICc < 2 (Supplementary Table S6). Model averaged parameter estimates indicated the percent developed land within 1000 m of ponds was a significant predictor of both baseline and agitation CORT release rates for ponds sampled in 2017 (Table 5). Baseline and agitation CORT release rates were both higher in ponds with more developed land within 1000 m (Fig. 4). In 2016, the marginal *R^2^* for the top model explaining baseline and agitation CORT release rates were 0.24 and 0.26, respectively. The conditional *R^2^* for these same models were 0.34 and 0.32, respectively. The marginal *R^2^* is calculated using only the fixed effects, whereas the conditional *R^2^* includes both fixed and random effects. Therefore, the inclusion of random effects and fixed effects explained more variation in CORT release rates than fixed effects alone. The difference between marginal and conditional *R^2^* values was greater for 2017, where the marginal *R^2^* for the top model explaining baseline CORT release rates was 0.23, and for agitation was 0.21, with the conditional *R^2^* values of 0.38 and 0.60, respectively. Average CORT release rates across both years were highest at the site in Lafayette Forest Wildlife Environmental Area (LF1; Fig. 5). Examining CORT release rates within each treatment for each pond indicated 40% (*N* = 4) of ponds sampled in 2016 and 50% (*N* = 6) of ponds sampled in 2017 did not show significant differences between baseline and agitation CORT release rates, though only one population (JC3) did not show a difference across both years (Supplementary Table S7; Fig. 5). However, not all ponds were resampled both years owing to a lack of tadpoles or complete loss of the pond in one case.

**Table 4:** Model averaged parameter estimates, unconditional SE and unconditional 95% CIs of environmental variables on baseline and agitation CORT release rates of *Pseudacris ornata* tadpoles from 10 sites sampled in 2016

Response	Predictor	Estimate	SE	Lower 95% CI	Upper 95% CI
Baseline CORT	PC1	0.1798	0.094	−0.0045	0.3641
	PC2	−0.2249	0.1167	−0.4537	0.0039
	MEM2	0.1981	0.1097	−0.0169	0.4131
	MEM3	0.0068	0.2336	−0.4511	0.4647
Agitation CORT	**PC1**	**0.1902**	**0.0447**	**0.1026**	**0.2778**
	**PC2**	**−0.2857**	**0.098**	**−0.4777**	**−0.0936**
	MEM2	0.0808	0.0814	−0.0787	0.2403
	**MEM3**	**−0.2326**	**0.0517**	**−0.334**	**−0.1313**
	Canopy500	−0.008	0.0059	−0.0194	0.0035

Only parameters from the top-ranking models (ΔAICc < 2) are included. Bolded terms indicate parameters used for inference whose 95% CIs do not overlap 0.

**Table 5:** Model averaged parameter estimates, unconditional SE and unconditional 95% CIs of environmental variables on baseline and agitation CORT release rates, mucosome function and bacterial diversity (Richness, Shannon and Simpson) of *P. ornata* tadpoles from 12 sites sampled in 2017

Response	Predictor	Estimate	SE	Lower 95% CI	Upper 95% CI
Baseline CORT	**Urban1000**	**0.117**	**0.037**	**0.046**	**0.189**
	PC2	0.082	0.103	−0.120	0.284
Agitation CORT	**Urban1000**	**0.126**	**0.053**	**0.022**	**0.231**
	PC1	−0.039	0.086	−0.206	0.129
Mucosome	PC1	−0.396	0.770	−1.905	1.113
	PC2	−2.279	1.320	−4.866	0.309
	Urban100	−0.095	0.076	−0.244	0.054
	Canopy500	−0.718	0.096	−0.259	0.116
Bacterial richness	**Landcover1000: Forest**	**386.824**	**32.137**	**323.836**	**449.811**
	**Landcover1000: Wetlands**	**−265.525**	**89.063**	**−440.085**	**−90.965**
	**Landcover1000: Shrub**	**−147.176**	**69.089**	**−282.588**	**−11.763**
	PC1	12.786	16.991	−20.516	46.087
Shannon diversity	**Urban500**	**0.060**	**0.027**	**0.006**	**0.114**
	**Canopy100**	**−0.012**	**−0.006**	**−0.023**	**−0.002**
	PC1	0.093	0.076	−0.056	0.241
	PC2	−0.167	0.096	−0.356	0.021
	**MEM2**	**0.251**	**0.110**	**0.036**	**0.466**
Simpson diversity	**MEM2**	**0.046**	**0.014**	**0.019**	**0.073**
	MEM3	0.024	0.031	−0.036	0.084

Only parameters from the top-ranking models (ΔAICc < 2) are included. Bolded terms indicate parameters used for inference whose 95% CIs do not overlap 0.

### Mucosome function

There was no significant difference in the mean mucosome function across all ponds (ANOVA: *F*_11,109_ = 0.476, *P* = 0.914; Supplementary Fig. S1). Levene’s test further determined no difference in the mean variance across all ponds (*W* = 1.19; *P* = 0.302). Mean *Bd* viability in the presence of the mucosome was 88.1%. Five candidate models (ΔAICc < 2) were selected from AICc model selection to explain the variation in mucosome function (Supplementary Table S6). These models included PC1, PC2, percent developed land within 100 m (Urban100) and the percent canopy within 500 m (Canopy500) as top predictors, though model averaged parameter estimates did not indicate any significant predictor (Table 5).

### Skin bacterial diversity

**Figure 6:**
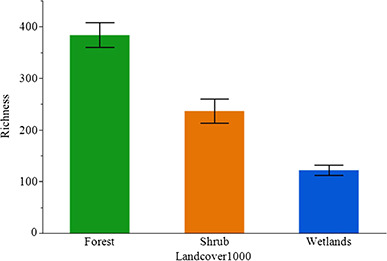
Mean ± SE skin bacterial richness of *P. ornata* tadpoles and the relationship with the dominant land cover type within 1000 m (Landcover1000) of each pond.

**Figure 7:**
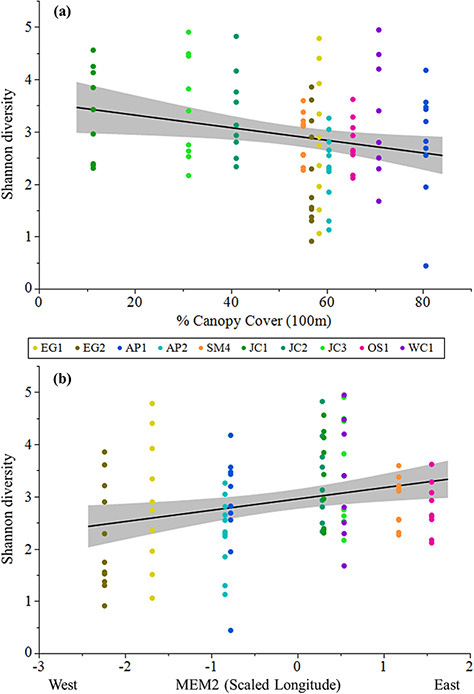
Shannon diversity of skin bacterial communities of *P. ornata* tadpoles varied significantly with (a) percent canopy cover within 100 m of each pond and (b) MEM2. Ponds: AP = Apalachicola National Forest, EG = Eglin Air Force Base, JC = Joseph W. Jones Ecological Research Center at Ichauway, LF = Lafayette Forest Wildlife Environmental Area, SM = St. Marks National Wildlife Refuge, OS = Orianne Society Preserve, WC = James W. Webb Wildlife Center.

**Figure 8:**
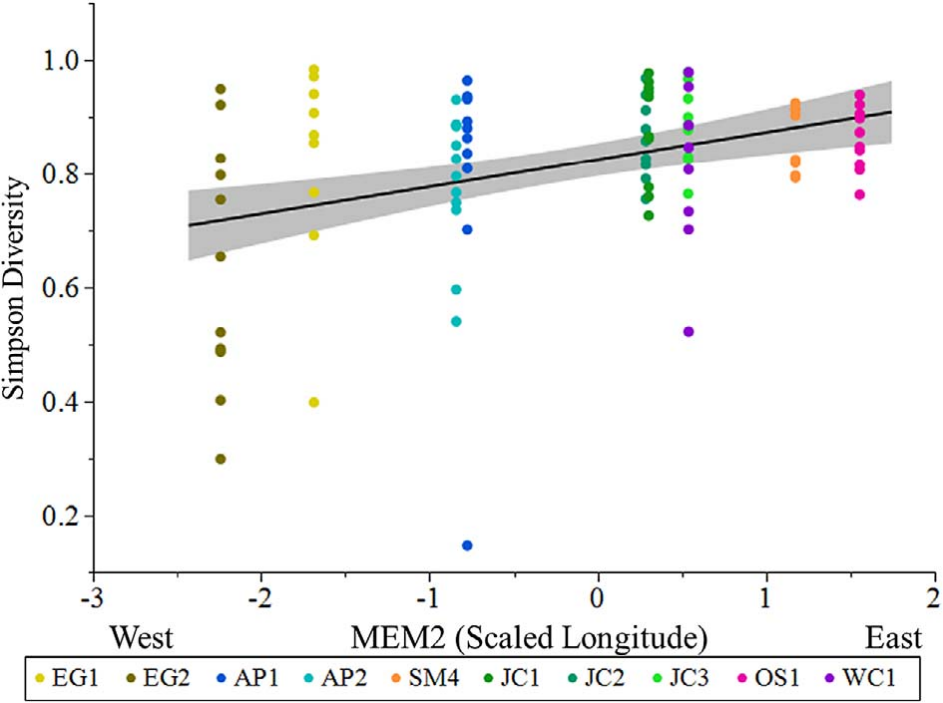
Simpson diversity of skin bacterial communities of *P. ornata* tadpoles varied significantly with MEM2. Ponds: AP = Apalachicola National Forest, EG = Eglin Air Force Base, JC = Joseph W. Jones Ecological Research Center at Ichauway, LF = Lafayette Forest Wildlife Environmental Area, SM = St. Marks National Wildlife Refuge, OS = Orianne Society Preserve, WC = James W. Webb Wildlife Center.

**Figure 9:**
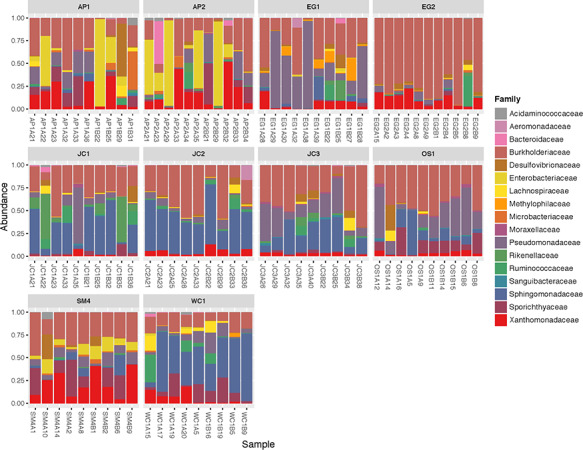
Mean relative abundance of bacterial families from the 50 most abundant taxa found on the skin of *P. ornata* tadpoles collected from 10 ponds in 2017. Ponds: AP = Apalachicola National Forest, EG = Eglin Air Force Base, JC = Joseph W. Jones Ecological Research Center at Ichauway, LF = Lafayette Forest Wildlife Environmental Area, SM = St. Marks National Wildlife Refuge, OS = Orianne Society Preserve, WC = James W. Webb Wildlife Center. Sample names on x axis are individual tadpole identifiers.

**Figure 10:**
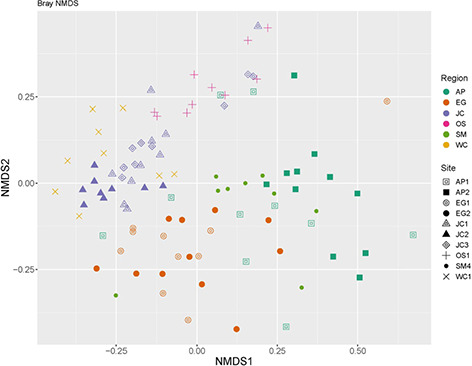
Ordination plot showing beta diversity (OTUs) of microbiota sampled from the skin of *P. ornata* tadpoles collected from 10 ponds in 2017 based on the Bray–Curtis method of NMDS. Colours represent Properties, and shapes represent Sites. Each point represents a single individual.

Alpha diversity of skin bacterial communities differed across Sites and Properties in both Richness and Shannon diversity (Richness, Site: *χ*^2^ = 42.80, df = 9, *P* < 0.001; Property: *χ*^2^ = 35.60, df = 5, *P* < 0.001; Shannon, Site: *F*_9,88_ = 2.32, *P* = 0.022; Property: *F*_5,92_ = 3.18, *P* = 0.011), but not in Simpson diversity (Simpson, Site: *χ*^2^ = 13.88, df = 9, *P* = 0.127; Property: *χ*^2^ = 7.61, df = 5, *P* = 0.179). Measures of diversity tended to be highest at sites within the Jones Center and lowest at sites within Eglin AFB, with the exception of Richness that was lowest within Apalachicola National Forest (Supplementary Table S8). Two candidate models (ΔAICc < 2) were selected to explain the variation in Richness across sites (Supplementary Table S6). Model averaged parameter estimates indicated dominant land cover within 1000 m of each pond was a significant predictor of bacterial richness (Table 5; Fig. 6), with Richness being highest in forest and lower in large wetlands and in ponds surrounded by shrubby vegetation. The top model explaining Richness had a marginal *R^2^* of 0.24 and a conditional *R^2^* of 0.36, indicating some of the variation was attributed to the random effect of Site. Six candidate models were selected to explain Shannon diversity (Supplementary Table S6). Model averaged parameter estimates indicated that the percent developed land within 500 m (Urban500; Supplementary Fig. S2) and the percent canopy cover within 100 m (Canopy100; Fig. 7a) were significant predictors of Shannon diversity (Table 5; Fig. 7a), with diversity being higher in ponds surrounded by more developed land and lower in ponds with more canopy cover. Additionally, Shannon diversity was higher as MEM2 increased (Table 5; Fig. 7b). The marginal and conditional *R^2^* for the top model explaining Shannon diversity were 0.07 and 0.10, respectively. Two candidate models were selected to explain Simpson diversity across ponds (Supplementary Table S6), with MEM2 (scaled longitude) being the only significant predictor (Table 5; Fig. 8). As MEM2 increased, Simpson diversity also increased. The marginal and conditional *R^2^* for the top model explaining Simpson diversity was 0.14. Prominent bacterial families included Burkholderiaceae, Sphingomonadaceae, Xanthomonadaceae, Pseudomonadaceae, Enterobacteriaceae and Desulfovibrionaceae (Fig. 9). The relative contribution of both Site and Property accounted for significant variation in microbiome beta diversity when analysed with a permutational ANOVA (Site: ADONIS *R*^2^ = 0.47, *P* = 0.001; Property: ADONIS *R*^2^ = 0.32, *P* = 0.001). All pairwise comparisons between Sites were significant (*P* < 0.05) except for the comparison of site AP1 to AP2 (*F* = 1.46, *P* = 0.144) and AP1 to SM4 (F = 1.58, *P* = 0.117; Supplementary Table S9). All pairwise comparisons between Properties were significant (*P* < 0.001) except for the comparison of the Apalachicola Property to St. Marks (*F* = 1.80, *P* = 0.066; Supplementary Table S10). An ordination plot based on the Bray–Curtis method of NMDS showed bacterial community similarities within Sites and across Properties (Fig. 10).

## Discussion

We found that both water quality and land cover characteristics were associated with increased CORT release rates and altered skin bacterial communities among populations of *P. ornata*. We found both baseline and agitation CORT profiles were linked to pond water quality, as well as the amount of developed land within 1000 m of ponds. These results indicate that environmental variation in two different settings—the aquatic and surrounding terrestrial habitats—operate to influence the physiology (GCs) of larval *P. ornata*. Similarly, pond water quality, land cover and spatial location of ponds were significant predictors of mucosome function and skin bacterial diversity, indicating environmental variation may affect larval *P. ornata* immune defences through changes in bacterial communities, though we did not see significant effects of these predictors directly on mucosome function. Therefore, perturbations and changes to environmental conditions at multiple spatial scales may significantly impact population health in this species. Given that some of these populations differed from year to year in their CORT profile, the impact of environmental variation—along with the ability to recover from stressor effects—likely varied over time, showing a need for continued monitoring (Blaustein *et al.*, 2011). Taken together, the effects of environmental variation on GC function and bacterial communities across years suggests that persistent deviations from optimum conditions may affect population health, thus setting the stage for population declines.

### Water-borne CORT release rates

Water quality variables and nearby urban development were important predictors of larval ornate chorus frog CORT profiles. In 2016, both baseline and agitation CORT release rates were higher in ponds with higher water temperature, conductivity and TDS, as shown by the relationship with PC1 and PC2, whereas the percent urban development within 1000 m was positively associated with CORT release rates in 2017. Additionally, agitation CORT release rates in 2016 were higher at lower MEM3 values, indicating ponds closest to each other were most similar and ponds at lower latitudes had higher CORT release rates, probably from the warmer water temperatures at the southernmost site (LF1). Similarly, studies involving salamanders also found elevated water-borne CORT release rates associated with higher temperatures (Novarro *et al.*, 2018; Millikin *et al.*, 2019) and higher conductivity (Chambers, 2011); although, conductivity did not significantly affect CORT in *Rana sylvatica* or *Hyla versicolor* tadpoles (Chambers, 2011). Both acute and chronic thermal stressors are associated with increased urinary CORT in *Rhinella marina*, the invasive cane toad (Narayan *et al.*, 2012; Narayan and Hero, 2014a and b). Elevated CORT can assist with energy mobilization and metabolic increases associated with higher temperatures but may also indicate stress (Chambers *et al.*, 2013; Narayan and Hero, 2014a and b; Novarro *et al.*, 2018; Millikin *et al.*, 2019). Several studies show increased developmental rate and body condition of tadpoles in warmer water (Schiesari, 2006; Reading, 2010), though stress is generally associated with lower body condition and reduced growth in amphibians (Glennemeier and Denver, 2002; Hu *et al.*, 2008; Denver, 2009; Janin *et al.*, 2011; Crespi and Warne, 2013). Therefore, higher water temperatures may be an environmental stressor for this winter-breeding amphibian, as warmer water temperatures may result in a shorter hydroperiod, which is a known environmental stressor itself (Denver, 1998; Gomez-Mestre *et al.*, 2013). Our results suggest environmental variation in water quality characteristics are important determinants of the CORT profiles of larval *P. ornata*, potentially affecting their physiological health.

Pond breeding frogs may be affected by habitat alterations at multiple scales due to their use of both ponds, as larvae and to breed, and terrestrial habitats as adults. Janin *et al.* (2011) found that body condition was lower and urinary CORT metabolites were higher in adult *Bufo* from ponds characterized by low forest availability and high fragmentation within 500 m, suggesting surrounding habitat affects amphibian health. We found that ponds with higher percentages of developed land within 1000 m were associated with higher water-borne CORT release rates in 2017. Gabor *et al.* (2018) found Jollyville Plateau salamanders (*Eurycea tonkawae*) had higher water-borne CORT release rates in more urbanized streams as indicated by greater percent impervious cover within the watershed. We also found that CORT release rates were highest in tadpoles from a pond within Lafayette Forest Wildlife Environmental Area (LF1), which had some of the warmest water temperatures and was dominated by shrubby vegetation, a result of infrequent prescribed fires. Of the properties sampled, this land was the most recently acquired and managed by the state of Florida (purchased in 2008, management began in 2010) with the first prescribed fires conducted in 2013 (N. Lambert, Florida Fish and Wildlife Conservation Commission, pers. comm.). Prescribed fire is vital to management of longleaf pine uplands and their embedded wetlands because it controls the growth of invasive, shrubby, mid-story vegetation such as saw palmetto (*Serenoa repens*) and gallberry (*Ilex coriacea*; Means, 2006; Noss, 2013; Enge *et al.*, 2014; Noss, 2018). These results suggest habitat quality at a larger scale may affect the CORT profile and subsequent physiological health of *P. ornata* tadpoles, whether by directly affecting larval health, or indirectly via increased stress on adults.

Elevated CORT in response to an acute stressor is indicative of a healthy endocrine response. Tadpoles that elevate CORT release in response to an acute agitation stressor indicate the HPI axis is still active and not dysregulated due to chronic stress. Therefore, the ability to mount a response to an acute stressor provides one component that can indicate a healthy population. Comparing the average baseline and agitation CORT release rates from each pond over both years showed four ponds in 2016 and six ponds in 2017 without a significant difference in their stress response (Fig. 5). The lack of a significant stress response in these populations suggests that these ponds may be suffering from chronic stress (Romero, 2004; Cyr and Romero, 2009). These populations may be able to recover if environmental suitability increases and/or if individuals show compensatory growth once they leave the pond and possibly find suitable habitat (Metcalfe and Monaghan, 2001). When individuals return as adults to the pond to lay eggs, their offspring may not show health effects if the water and environmental quality have improved. However, stressful conditions during larval development may have negative post-metamorphic carry-over effects on juvenile and adult frogs (Crespi and Warne, 2013). The populations that did not show a healthy CORT response may fail to recover when exposed to consecutive years of low environmental quality, or if the stressful conditions experienced during the larval stage have carry-over effects in the adults. Populations of *P. ornata* in the peninsular region of Florida are declining and undergoing local extirpations (Enge *et al.*, 2014). We were unable to find *P. ornata* tadpoles (nor measure CORT) in this region, and it is possible that these declines may be the consequence of an inability to recover from past harsh environmental conditions (or repeated harsh conditions) such as the drought (and associated warmer temperatures) experienced by this region from 2006 to 2008 and from 2010 to 2012 (Enge *et al.*, 2014).

Even though our results indicate environmental variation at multiple spatial and environmental scales (i.e. water quality vs. terrestrial habitat) impact the CORT profiles of larval *P. ornata*, random effects in the models (as indicated by the conditional *R^2^*) accounted for some of the variation in CORT release rates. This suggests additional variability between ponds within each property associated with unmeasured factors. The difference between the marginal and conditional *R^2^* was greater in 2017, indicating more variation in the CORT release rates between these sites was not accounted for by the predictors. This is likely due to natural differences in pond characteristics (i.e. size, depth, hydroperiod) and other habitat factors not examined. This variation contributes to the uncertainty that enshrouds management decisions for *P. ornata* and ecologically similar species. However, the consequences of development and water quality on the health of *P. ornata*, indicated in this study, lends additional support for the conservation and appropriate management of longleaf pine forests, including frequent prescribed fire during the growing season, to maintain high-quality pine savannahs for this and other longleaf pine endemic species (Means, 2006; Noss, 2013; Enge *et al.*, 2014; Noss, 2018).

### Mucosome function

The amphibian mucosome, including both skin secretions and microbial communities, are important factors in amphibian health and immunity. The ability of the mucosome to fight infection has been used as a holistic measure of amphibian skin defences (Woodhams *et al.*, 2014). We found no differences in the mean mucosome function across ponds, though the *Bd* cell viability within a pond was quite variable. Additionally, there were no significant predictors of mucosome function in our models, though water quality and landscape variables were included in the top models. These results indicate no difference in the bacterial and mucosal defences across ponds. In this study, mucosome function only reduced *Bd* cell viability to 88.1%, indicating this species may be susceptible to *Bd* infection. Indeed, Gervasi *et al.* (2017) found that *P. ornata* had the highest average *Bd* infection load of 20 species studied and a high rate of mortality following infection. Similarly, Horner *et al.* (2017) found *P. ornata* had a high infection intensity and a high *Bd* prevalence across ponds. However, due to logistics, we were unable to use the strain of *Bd* to which these frogs are naturally exposed, which may be why we did not see any difference in mucosome function across ponds. Woodhams *et al.* (2014) found a significant correlation between mucosome function and both field and laboratory *Bd* infection prevalence for Swiss and European frogs (for a Swiss *Bd* strain); thus, future studies could repeat exposure experiments using local isolates.

### Skin bacterial diversity

Alpha and beta skin bacterial diversity varied across both Sites and Properties. Primary predictors of bacterial diversity at each pond were the dominant land cover type within 1000 m, the percent developed land within 500 m, the percent canopy cover within 100 m and the spatial location of ponds. All alpha diversity indices were highest at sites located within the Jones Center property, and these wetlands were the largest sampled and surrounded by mature longleaf pine forest with short-interval prescribed fires to maintain high-quality pine savannah. Even though a site in Apalachicola National Forest (AP2) had the lowest observed Richness, both Shannon and Simpson diversity indices were lowest within the Eglin Air Force Base (EG) property. The ponds sampled at Eglin were along a powerline right-of-way and thus were composed of more disturbed habitat. Variation in skin bacterial communities are generally associated with pond characteristics including conductivity, pH, temperature, dissolved oxygen and precipitation (Kueneman *et al.*, 2013; Krynak *et al.*, 2015, 2016; Varela *et al.*, 2018). The significant association of bacterial diversity with land cover suggests landscape-scale features also affect bacterial communities. Bacterial richness, the number of species present, was positively associated with ponds surrounded by forest. Interestingly, Shannon diversity, a measure of the abundance and variety of bacteria present, was higher in ponds with more developed land within 500 m and with less canopy cover within 100 m. Enge *et al.* (2014) found that most wetlands inhabited by *P. ornata* lacked a canopy or had an open canopy that allowed growth of grasses during dry down periods, a result of short-interval fire management. Our results suggest these open canopy ponds with greater environmental variation may have higher bacterial diversity.

In southeastern USA, land-use conversion, fire suppression and droughts are important factors in the decline of amphibian species, especially those reliant on the savannah-like longleaf pine-wiregrass (*P. palustris-Aristida* spp.) ecosystem. In addition to its control of invasive shrubs and hardwoods, summer growing-season fire, when ponds are typically dry, is required to maintain grasses and sedges in ephemeral wetlands used by those pond-breeding amphibians that are endemic to this ecosystem (Noss, 2013, 2018). Within the pine savannahs of the southeastern Coastal Plain, a short-interval fire regime maintains herbaceous vegetation, reduces canopy cover in breeding ponds and increases amphibian abundance and diversity (Noss, 2013, 2018). Our findings that landcover type, the percent developed land and increased canopy cover were associated with altered skin bacterial diversity indicate environmental variation may also contribute to changes in skin bacterial diversity of larval *P. ornata*. These effects may ultimately impact the immune defences of species like *P. ornata* that are specialists of the longleaf pine ecosystem.

The sole significant predictor of Simpson diversity, and an additional significant predictor of Shannon diversity, was the spatial MEM axis MEM2 (scaled longitude). The eigenvalue for this axis was −0.78, indicating autocorrelation among sites at a fine spatial scale. This autocorrelation indicates both Shannon and Simpson diversities are most similar in nearby ponds, which is expected, and that longitude had an effect on the diversity observed. Our results suggest ponds sampled in the western portion of the range had lower Shannon and Simpson bacterial diversity than ponds sampled in the eastern portion of the range. The more similar diversity among ponds in close proximity to one another is also suggested in the results of our distance-based ordination plot of bacterial diversity (Fig. 10). However, the pairwise comparison of beta diversity between sites showed that all comparisons between sites except two showed significant differences (Table S8) and all comparisons between properties were significantly different except for those that were geographically the closest (Apalachicola National Forest and St. Marks National Wildlife Refuge; Table S9). As we have seen, environmental characteristics can influence bacterial diversity. Additionally, maintaining high-quality ponds and wetlands across a species range can aid in establishing a higher bacterial diversity. Together, these results suggest site location and landscape characteristics around ponds play an important role in the skin bacterial community composition, which can affect immune defences, ultimately influencing the health of these larval amphibians.

Though bacterial communities are known to vary across sites and even among species within the same site (see Belden *et al.*, 2015), some bacterial families have high proportions of *Bd* inhibitory isolates. Bletz *et al.* (2017b) examined skin bacteria from Madagascar amphibians and found three families with 75% or more of the isolates having inhibitory effects on *Bd* infection, suggesting these may be good candidates for broad probiotic treatment. These three families (Enterobacteriaceae, Pseudomonadaceae and Xanthamonadaceae) were also well represented in our site diversity; although, *P. ornata* have high *Bd* prevalence (Gervasi *et al.*, 2017) and the mucosome function in our study only reduced *Bd* cell viability to ~88% (see Mucosome function under Results above), suggesting other factors may be contributing to the infection of this species. Continued research should consider the skin bacterial community to aid in developing probiotics to fight known pathogens such as *B. dendrobatidis* and *B. salamandrivorans* (Woodhams *et al.*, 2016).

## Conclusions

Amphibian populations have been declining rapidly worldwide, and this decline is likely tied to their need for multiple habitats and their susceptibility to environmental changes (Stuart *et al.*, 2004; Becker *et al.*, 2007). There is a growing need to monitor population health and factors that influence their health to provide early warning signs for proactive management decisions. Our results suggest the conservation of *P. ornata* could be enhanced by management actions that address water quality and forest composition to maintain high-quality aquatic and terrestrial habitat. Natural resource managers could consider habitat quality at multiple spatial scales to best manage larval amphibian populations and the bacterial communities present. By examining baseline and stress (agitation) induced CORT profiles and other health metrics across locations and for several years in succession, researchers and managers may be able to use this method proactively to identify populations at increased risk of declines to focus management efforts. Additionally, little is known about the adult life history of this cryptic and fossorial species (Brown and Means, 1984; Bartlett and Bartlett, 2011), warranting additional research and implementing management practices that maintain suitable habitat for both aquatic and terrestrial life stages, as landscape-level factors may have impacts in aquatic larvae directly, as well as indirectly by affecting adults. By examining environmental variables, the CORT profiles and skin bacterial communities of populations of *P. ornata* across the range of the species, we identified factors at multiple spatial scales that affect population health. Though some populations showed an ability to mount a CORT response in different years, without habitat remediation, other populations may no longer be able to recover from changes in environmental conditions.

## Funding

This work was supported by a cooperative agreement between the U.S. Geological Survey and Texas State University (G15AC00457 to C.R.G. and S.C.W.) and start-up funds from Texas State University to D.R.

## Supplementary Material

Supplementary_Material_coaa047

## References

[ref1] Barnhardt KL (2018) From symbionts to pathogens: interactions within the amphibian skin mucosome. Master’s thesis. University of Massachusetts, Boston, ProQuest Dissertations Publishing (10788783).

[ref2] Bartlett RD , Bartlett PP (2011) Florida’s Frogs, Toads, and Other Amphibians. University Press of Florida, Gainesville, pp. 95–96.

[ref3] Barton K (2018) MuMin: multi-model inference. R Package Version 1.1. https://CRAN.R-project.org/package=MuMIn.

[ref4] Baugh AT , Bastien B, Still MB, Stowell N (2018) Validation of water-borne steroid hormones in a tropical frog (*Physalaemus pustulosus*). Gen Comp Endocrinol 261: 67–80. 29397994 10.1016/j.ygcen.2018.01.025

[ref5] Becker CG , Fonseca CR, Haddad CFB, Batista RF, Prado PI (2007) Habitat split and the global decline of amphibians. Science 318: 1775–1777. 18079402 10.1126/science.1149374

[ref6] Becker CG , Rodriguez D, Lambertini C, Toledo LF, Haddad CFB (2015) Historical dynamics of *Batrachochytrium dendrobatidis* in Amazonia. Ecography 38: 001–007.

[ref7] Belden LK , Hughey MC, Rebollar EA, Umile TP, Loftus SC, Burzynski EA, Minbiole KPC, House LL, Jensen RV, Becker MH et al. (2015) Panamanian frog species host unique skin bacterial communities. Front Microbiol 6: 1171. 26579083 10.3389/fmicb.2015.01171 PMC4621460

[ref9] Blaustein AR , Han BA, Relyea RA, Johnson PTJ, Buck JC, Gervasi SS, Kats LB (2011) The complexity of amphibian population declines: understanding the role of cofactors in driving amphibian losses. Ann N Y Acad Sci 1223: 108–119. 21449968 10.1111/j.1749-6632.2010.05909.x

[ref10] Bletz MC , Perl RGB, Bobowski BTC, Japke LM, Tebbe CC, Dohrmann AB, Bhuju S, Geffers R, Jarek M, Vences M (2017a) Amphibian skin microbiota exhibits temporal variation in community structure but stability of predicted *Bd*-inhibitory function. ISME J 11: 1521–1534. 28387770 10.1038/ismej.2017.41 PMC5520157

[ref11] Bletz MC , Myers J, Woodhams DC, Rabemananjara FCE, Rakotonirina A, Weldon C, Edmonds D, Vences M, Harris RN (2017b) Estimating herd immunity to amphibian chytridiomycosis in Madagascar based on the defensive function of amphibian skin bacteria. Front Microbiol 8: 1751. 28959244 10.3389/fmicb.2017.01751 PMC5604057

[ref12] Breuner CW , Delehanty B, Boonstra R (2013) Evaluating stress in natural populations of vertebrates: total CORT is not good enough. Funct Ecol 27: 24–36.

[ref13] Brown LE , Means DB (1984) Fossorial behaviour and ecology of the chorus frog *Pseudacris ornata*. Amphibia-Reptilia 5: 261–273.

[ref14] Brown ME , Walls SC (2013) Variation in salinity tolerance among larval anurans: implications for community composition and the spread of an invasive, non-native species. Copeia 2013: 543–551.

[ref15] Brook BW , Sodhi NS, Bradshaw CJA (2008) Synergies among extinction drivers under global climate change. Trends Ecol Evol 23: 453–460. 18582986 10.1016/j.tree.2008.03.011

[ref16] Burraco P , Gomez-Mestre I (2016) Physiological stress responses in amphibian larvae to multiple stressors reveal marked anthropogenic effects even below lethal levels. Physiol Biochem Zool 89: 462–472. 27792531 10.1086/688737

[ref17] Callahan BJ , McMurdie PJ, Rosen MJ, Han AW, Johnson AA, Holmes SP (2016) DADA2: high-resolution sample inference from Illumina amplicon data. Nat Methods 13: 581–583 Bioconductor Version 1.8.0. 27214047 10.1038/nmeth.3869 PMC4927377

[ref18] Caporaso JG , Lauber CL, Walters WA, Berg-Lyons D, Lozupone CA, Turnbaugh PJ, Fierer N, Knight R (2011) Global patterns of 16S rRNA diversity at a depth of millions of sequences per sample. Proc Natl Acad Sci U S A 108: 4516–4522. 20534432 10.1073/pnas.1000080107 PMC3063599

[ref20] Ceballos G , Ehrlich PR, Dirzo R (2017) Biological annihilation via the ongoing sixth mass extinction signaled by vertebrate population losses and declines. Proc Natl Acad Sci U S A 114: E6089–E6086. 28696295 10.1073/pnas.1704949114 PMC5544311

[ref21] Chambers DL (2011) Increased conductivity affects corticosterone levels and prey consumption in larval amphibians. J Herpetol 45: 219–223.

[ref22] Chambers DL , Wojdak JM, Du P, Belden LK (2013) Pond acidification may explain differences in corticosterone among salamander populations. Physiol Biochem Zool 86: 224–232. 23434782 10.1086/669917

[ref23] Clarke G , Stilling RM, Kennedy PJ, Stanton C, Cryan JF, Dinan TG (2014) Minireview: gut microbiota: the neglected endocrine organ. Mol Endocrinol 28: 1221–1238. 24892638 10.1210/me.2014-1108 PMC5414803

[ref24] Crespi EJ , Warne RW (2013) Environmental conditions experienced during the tadpole stage alter post-metamorphic glucocorticoid response to stress in an amphibian. Integr Comp Biol 53: 989–1001. 23922274 10.1093/icb/ict087

[ref25] Cyr NE , Romero LM (2009) Identifying hormonal habituation in field studies of stress. Gen Comp Endocrinol 161: 295–303. 19523375 10.1016/j.ygcen.2009.02.001

[ref26] Dantzer B , Fletcher QE, Boonstra R, Sheriff MJ (2014) Measures of physiological stress: a transparent or opaque window into the status, management and conservation of species? Conserv Physiol 2: cou023. 10.1093/conphys/cou023. PMC4732472 27293644

[ref27] Daszak P , Scott DE, Kilpatrick AM, Faggioni C, Gibbons JW, Porter D (2005) Amphibian population declines at Savannah River Site are linked to climate, not chytridiomycosis. Ecology 86: 3232–3237.

[ref29] Denver RJ (1998) Hormonal correlates of environmentally induced metamorphosis in the Western spadefoot toad, *Scaphiopus hammondii*. Gen Comp Endocrinol 110: 326–336. 9593653 10.1006/gcen.1998.7082

[ref30] Denver RJ (2009) Stress hormones mediate environment-genotype interactions during amphibian development. Gen Comp Endocrinol 164: 20–31. 19393659 10.1016/j.ygcen.2009.04.016

[ref31] Dhabhar FS , McEwen BS (1999) Enhancing versus suppressive effects of stress hormones on skin immune function. Proc Natl Acad Sci U S A 96: 1059–1064. 9927693 10.1073/pnas.96.3.1059 PMC15350

[ref32] Dorcas M , Gibbons W (2008) Frogs and Toads of the Southeast. University of Georgia Press, Athens, pp. 62–64.

[ref33] Dray S , Bauman D, Blanchet G, Borcard D, Clappe S, Guenard G, Jombart T, Larocque G, Legendre P, Madi N et al. (2018) Adespatial: multivariate multiscale spatial analysis. R Package Version 0.3.2. https://CRAN.R-project.org/package=adespatial.

[ref35] Ellis T , James JD, Stewart C, Scott AP (2004) A non-invasive stress assay based upon measurement of free cortisol released into the water by rainbow trout. J Fish Biol 65: 1233–1252.

[ref36] Enge KM , Farmer AL, Mays JD, Castellon TD, Hill EP, Moler PE (2014) Survey of Winter-Breeding Amphibian Species. Final Report. Florida Fish and Wildlife Conservation Commission. Fish and Wildlife Research Institute, Wildlife Research Section, Gainesville, p. 136

[ref38] Fahrig L (2003) Effects of habitat fragmentation on biodiversity. Annu Rev Ecol Evol Syst 34: 487–515.

[ref39] Farrer RA , Weinert LA, Bielby J, Garner TWJ, Balloux F, Clare F, Bosch J, Cunningham AA, Weldon C, du Preez H et al. (2011) Multiple emergences of genetically diverse amphibian-infecting chytrids include a globalized hypervirulent recombinant lineage. Proc Natl Acad Sci U S A 108: 18732–18736. 22065772 10.1073/pnas.1111915108 PMC3219125

[ref40] Foden WB , Butchart SHM, Stuart SN, Vie J, Akcakaya HR, Angulo A, DeVantier LM, Gutsche A, Turak E, Cao E et al. (2013) Identifying the world’s most climate change vulnerable species: a systematic trait-based assessment of all birds, amphibians, and corals. PLoS One 8: e65427. 23950785 10.1371/journal.pone.0065427 PMC3680427

[ref19] Forsburg ZR , Goff CB, Perkins HR, Robicheaux JR, Almond GF, Gabor CR (2019) Validation of water-borne cortisol and corticosterone in tadpoles: Recovery rate from an acute stressor, repeatability, and evaluating rearing methods. Gen Comp Endocrinol 281: 145–152. 31199927 10.1016/j.ygcen.2019.06.007

[ref41] Gabor CR , Bosch J, Fries JN, Davis DR (2013a) A non-invasive water-borne hormone assay for amphibians. Amphibia-Reptilia 34: 151–162.

[ref42] Gabor CR , Fisher MC, Bosch J (2013b) A non-invasive stress assay shows that tadpole populations infected with *Batrachochytrium dendrobatidis* have elevated corticosterone levels. PLoS One 8: e56054. 23418508 10.1371/journal.pone.0056054 PMC3572145

[ref43] Gabor CR , Fisher MC, Bosch J (2015) Elevated corticosterone levels and changes in amphibian behavior are associated with *Batrachochytrium dendrobatidis* (*Bd*) infection and *Bd* lineage. PLoS One 10: e0122685. 25893675 10.1371/journal.pone.0122685 PMC4404099

[ref44] Gabor CR , Zabierek KC, Kim DS, Alberici da Barbiano L, Mondelli MJ, Bendik NF, Davis DR (2016) A non-invasive water-borne assay of stress hormones in aquatic salamanders. Copeia 104: 172–181.

[ref45] Gabor CR , Davis DR, Kim DS, Zabierek KC, Bendik NF (2018) Urbanization is associated with elevated corticosterone in Jollyville plateau salamanders. Ecol Indic 85: 229–235.

[ref46] Gastwirth JL , Gel YR, Hui WLW, Lyubchich V, Miao W, Noguchi K (2017) Lawstat: tools for biostatistics, public policy, and law. R Package Version 3.2. https://CRAN.R-project.org/package=lawstat.

[ref47] Gervasi SS , Stephens PR, Hua J, Searle CL, Yang Xie G, Urbina J, Olson DH, Bancroft BA, Weis V, Hammond JI et al. (2017) Linking ecology and epidemiology to understand predictors of multi-host responses to an emerging pathogen, the amphibian chytrid fungus. PLoS One 12: e0167882. 28095428 10.1371/journal.pone.0167882 PMC5240985

[ref48] Gilbert JA , Jansson JK, Knight R (2014) The Earth Microbiome project: successes and aspirations. BMC Biol 12: 69. 25184604 10.1186/s12915-014-0069-1 PMC4141107

[ref28] Glennemeier KA , Denver RJ (2002) Developmental changes in interrenal responsiveness in anuran amphibians. Integr Comp Biol 42: 565–573. 21708752 10.1093/icb/42.3.565

[ref34] Gomez-Mestre I , Kulkarni S, Buchholz DR (2013) Mechanisms and consequences of developmental acceleration in tadpoles responding to pond drying. PLoS One 8: e84266. 24358352 10.1371/journal.pone.0084266 PMC3865288

[ref49] Gorman TA , Haas CA, Himes JG (2013) Evaluating methods to restore amphibian habitat in fire-suppressed pine flatwoods wetlands. Fire Ecol 9: 96–109.

[ref37] Gosner KL (1960) A simplified table for staging anuran embryos and larvae with notes on identification. Herpetologica 16: 183–190.

[ref50] Harris RN , Brucker RM, Walke JB, Becker MH, Schwantes CR, Flaherty DC, Lam BA, Woodhams DC, Briggs CJ, Redenburg VT et al. (2009) Skin microbes on frogs prevent morbidity and mortality caused by a lethal skin fungus. ISME J 3: 818–824. 19322245 10.1038/ismej.2009.27

[ref51] Hayes TB , Falso P, Gallipeau S, Stice M (2010) The cause of global amphibian declines: a developmental endocrinologist’s perspective. J Exp Biol 213: 921–933. 20190117 10.1242/jeb.040865 PMC2829317

[ref52] Hernandez-Gomez O , Hoverman JT, Williams RN (2017) Cutaneous microbial community variation across populations of Eastern Hellbenders (*Cryptobranchus alleganiensis alleganiensis*). Front Microbiol 8: 1379. 28785252 10.3389/fmicb.2017.01379 PMC5519570

[ref53] Hijmans RJ (2017) Geosphere: spherical trigonometry. R Package Version 1.7. https://CRAN.R-project.org/package=geosphere.

[ref54] Homan RN , Regosin JV, Rodrigues DM, Reed JM, Windmiller BS, Romero LM (2003) Impacts of varying habitat quality on the physiological stress of spotted salamanders (*Ambystoma maculatum*). Anim Conserv 6: 11–18.

[ref55] Horner AA , Hoffman EA, Tye MR, Hether TD, Savage AE (2017) Cryptic chytridiomycosis linked to climate and genetic variation in amphibian populations of the southeastern United States. PLoS One 12: e0175843. 28448517 10.1371/journal.pone.0175843 PMC5407605

[ref8] Hu F , Crespi EJ, Denver RJ (2008) Programming neuroendocrine stress axis activity by exposure to glucocorticoids during postembryonic development of the frog, *Xenopus laevis*. Endocrinol 149: 5470–5481. 10.1210/en.2008-0767 18653715

[ref56] IUCN (2019) The IUCN Red List of Threatened Species. Version 2019.2. http://www.iucnredlist.org (last accessed 15 November 2019).

[ref57] Jani AJ , Briggs CJ (2018) Host and aquatic environment shape the amphibian skin microbiome but effects on downstream resistance to the pathogen *Batrachochytrium dendrobatidis* are variable. Front Microbiol 9: 487. 29619014 10.3389/fmicb.2018.00487 PMC5871691

[ref58] Janin A , Lena J, Joly P (2011) Beyond occurrence: body condition and stress hormone as integrative indicators of habitat availability and fragmentation in the common toad. Biol Conserv 144: 1008–1016.

[ref59] Janin A , Lena J, Deblois S, Joly P (2012) Use of stress-hormone levels and habitat selection to assess functional connectivity of a landscape for an amphibian. Conserv Biol 26: 923–931. 22891816 10.1111/j.1523-1739.2012.01910.x

[ref60] Jeffrey JD , Hasler CT, Chapman JM, Cooke SJ, Suski CD (2015) Linking landscape-scale disturbances to stress and condition of fish: implications for restoration and conservation. Integr Comp Biol 55: 618–630. 25931612 10.1093/icb/icv022

[ref62] Kindermann C , Narayan EJ, Hero JM (2017) Does physiological response to disease incur cost to reproductive ecology in a sexually dichromatic amphibian species? Comp Biochem Physiol A Mol Integr Physiol 203: 220–226. 27712921 10.1016/j.cbpa.2016.09.019

[ref64] Kohl KD , Yahn J (2016) Effects of environmental temperature on the gut microbial communities of tadpoles. Environ Microbiol 18: 1561–1565. 26940397 10.1111/1462-2920.13255

[ref65] Krynak KL , Burke DJ, Bernard MF (2015) Larval environment alters amphibian immune defenses differently across life stages and populations. PLoS One 10: e0130383. 26107644 10.1371/journal.pone.0130383 PMC4479591

[ref66] Krynak KL , Burke DJ, Bernard MF (2016) Landscape and water characteristics correlate with immune defense traits across Blanchard’s cricket frog (*Acris blanchardi*) populations. Biol Conserv 193: 153–167.

[ref67] Kueneman JG , Wegener Parfrey W, Woodhams DC, Archer HM, Knight R, McKenzie VJ (2013) The amphibian skin-associated microbiome across species, space, and life history stages. Mol Ecol 23: 1238–1250. 24171949 10.1111/mec.12510

[ref68] Mantyka-Pringle CS , Martin TG, Rhodes JR (2012) Interactions between climate and habitat loss effects on biodiversity: a systematic review and meta-analysis. Glob Chang Biol 18: 1239–1252.

[ref69] Marshall TL , Baca CR, Correa DT, Forstner MRJ, Hahn D, Rodriguez D (2019) Genetic characterization of chytrids isolated from larval amphibians collected in central and east Texas. Fungal Ecol 39:55–62.

[ref70] Martinez Arbizu P (2019) pairwiseAdonis: pairwise multilevel comparison using Adonis. R Package Version 0.3. https://github.com/pmartinezarbizu/pairwiseAdonis/.

[ref71] Mazerolle M (2019) AICcmodavg: model selection and multimodel inference based on (Q)AIC(c). R Package Version 2.2.2. https://CRAN.R-project.org/package=AICcmodavg.

[ref72] McEwen BS , Wingfield JC (2003) The concept of allostasis in biology and biomedicine. Horm Behav 43: 2–15. 12614627 10.1016/s0018-506x(02)00024-7

[ref73] McKinney ML (2002) Urbanization, biodiversity, and conservation. Bioscience 52: 883–890.

[ref74] McMurdie PJ , Holmes S (2013) Phyloseq: an R package for reproducible interactive analysis and graphics of microbiome census data. PLoS One 8: e61217 Bioconductor Version 1.24.2. 23630581 10.1371/journal.pone.0061217 PMC3632530

[ref75] Means DB , Simberloff D (1987) The peninsula effect: habitat-correlated species decline in Florida’s herpetofauna. J Biogeogr 14: 551–568.

[ref76] Means DB (2006) Vertebrate Faunal Diversity of Longleaf Pine Ecosystems. In S Jose, EJ Jokela, DL Miller, eds, The Longleaf Pine Ecosystem: Ecology, Silviculture, and Restoration. Springer, New York,pp. 157–213.

[ref77] Metcalfe NB , Monaghan P (2001) Compensation for a bad start: grow now, pay later? Trends Ecol Evol 16: 254–260. 11301155 10.1016/s0169-5347(01)02124-3

[ref78] Meybeck M (2004) The global change of continental aquatic systems: dominant impacts of human activities. Water Sci Technol 49: 73–83. 15195419

[ref79] Millikin AR , Woodley SK, Davis DR, Anderson JT (2019) Habitat characteristics in created vernal pools impact spotted salamander water-borne corticosterone levels. Wetlands 39: 803–814.

[ref80] Narayan EJ , Cockrem JF, Hero JM (2012) Effects of temperature on urinary corticosterone metabolite responses to short-term capture and handling stress in the cane toad (*Rhinella marina*). Gen Comp Endocrinol 178: 301–305. 22728158 10.1016/j.ygcen.2012.06.014

[ref81] Narayan EJ , Hero JM (2014a) Acute thermal stressor increases glucocorticoid response but minimized testosterone and locomotor performance in the cane toad (*Rhinella marina*). PLoS One 9: e92090. 24643017 10.1371/journal.pone.0092090 PMC3958476

[ref82] Narayan EJ , Hero JM (2014b) Repeated thermal stressor causes chronic elevation of baseline corticosterone and suppresses the physiological endocrine sensitivity to acute stressor in the cane toad (*Rhinella marina*). J Therm Biol 41: 72–76. 24679975 10.1016/j.jtherbio.2014.02.011

[ref83] Noss RF (2013) Forgotten Grasslands of the South: Natural History and Conservation. Island Press, Washington

[ref84] Noss RF (2018) Fire Ecology of Florida and the Southeastern Coastal Plain. University Press of Florida, Gainesville

[ref85] Novarro AJ , Gabor CR, Goff CB, Mezebish TD, Thompson LM, Grayson KL (2018) Physiological responses to elevated temperature across the geographic range of a terrestrial salamander. J Exp Biol 221: jeb178236. 30072387 10.1242/jeb.178236

[ref86] Oksanen J , Blanchet FG, Friendly M, Kindt R, Legendre P, McGlinn D, Minchin PR, O’Hara PR, Simpson GL, Solymos P et al. (2019) Vegan: community ecology package. R Package Version 2.4. https://CRAN.R-project.org/package=vegan.

[ref87] Peterson JD , Steffen JE, Reinert LK, Cobine PA, Appel A, Rollins-Smith L, Mendonca MT (2013) Host stress response is important for the pathogenesis of the deadly amphibian disease, Chytridiomycosis, in *Litoria caerulea*. PLoS One 8: e62146. 23630628 10.1371/journal.pone.0062146 PMC3632538

[ref88] Pinheiro J , Bates D (2018) Nlme: linear and nonlinear mixed effects models. R Package Version 3.1.137. https://CRAN.R-project.org/package=nlme.

[ref89] Powell R , Conant R, Collins JT (2016) Peterson Field Guide to Reptiles and Amphibians of Eastern and Central North America, Ed4th. Houghton Mifflin Harcourt, Boston

[ref90] R Core Team (2018) R: A Language and Environment for Statistical Computing. R Foundation for Statistical Computing, Vienna, Austria, https://www.R-project.org/ (last accessed 2019).

[ref61] Reading CJ (2010) The impact of environmental temperature on larval development and metamorph body condition in the common toad, *Bufo bufo*. Amphibia-Reptilia 31: 483–488.

[ref91] Rollins-Smith LA (2017) Amphibian immunity—stress, disease, and climate change. Dev Comp Immunol 66: 111–119. 27387153 10.1016/j.dci.2016.07.002

[ref92] Rollins-Smith LA , Ramsey JP, Pask JD, Reinert LK, Woodhams DC (2011) Amphibian immune defenses against chytridiomycosis: impacts of changing environments. Integr Comp Biol 51: 552–562. 21816807 10.1093/icb/icr095

[ref93] Romero LM (2004) Physiological stress in ecology: lessons from biomedical research. Trends Ecol Evol 19: 245–255. 16701264 10.1016/j.tree.2004.03.008

[ref94] Romero LM , Dickens MJ, Cyr NE (2009) The reactive scope model—a new model integrating homeostasis, allostasis, and stress. Horm Behav 55: 375–389. 19470371 10.1016/j.yhbeh.2008.12.009

[ref95] Rosband WS (1997) ImageJ. Version 1.52a. http://imagej.nih.gov/ij/.

[ref96] Rosenblum EB , James TY, Zamudio KR, Poorten TJ, Ilut D, Rodriguez D, Eastman JM, Richards-Hrdlicka K, Joneson S, Jenkinson TS et al. (2013) Complex history of the amphibian-killing chytrid fungus revealed with genome resequencing data. Proc Natl Acad Sci U S A 110: 9385–9390. 23650365 10.1073/pnas.1300130110 PMC3677446

[ref97] Sapolsky RM , Romero LM, Munck AU (2000) How do glucocorticoids influence stress responses? Integrating permissive, suppressive, stimulatory, and preparative actions. Endocr Rev 21: 55–89. 10696570 10.1210/edrv.21.1.0389

[ref63] Schiesari L (2006) Pond canopy cover: a resource gradient for anuran larvae Freshw Biol 51: 412–423.

[ref98] Segan DB , Murray KA, Watson JEM (2016) A global assessment of current and future biodiversity vulnerability to habitat loss-climate change interactions. Glob Ecol Conserv 5: 12–21.

[ref99] Semlitsch RD , Scott DE, Pechmann JHK, Gibbons JW (1996) Structure and dynamics of an amphibian community: evidence from a 16-year study of a natural pond. In ML Cody, JA Smallwood, eds, Long-Term Studies of Vertebrate Communities. Academic Press, San Diego, CA, pp. 217–248

[ref100] Sheriff MJ , Dantzer B, Delehanty B, Palme R, Boonstra R (2011) Measuring stress in wildlife: techniques for quantifying glucocorticoids. Oecologia 166: 869–887. 21344254 10.1007/s00442-011-1943-y

[ref101] Skelly DK , Halverson MA, Freidenburg LK, Urban MC (2005) Canopy closure and amphibian diversity in forested wetlands. Wetl Ecol Manag 13: 261–268.

[ref102] Stuart SN , Chanson JS, Cox NA, Young BE, Rodrigues ASL, Fischman DL, Waller RW (2004) Status and trends of amphibian declines and extinctions worldwide. Science 306: 1783–1785. 15486254 10.1126/science.1103538

[ref103] Varela BJ , Lesbarreres D, Ibanez R, Green DM (2018) Environmental and host effects on skin bacterial community composition in Panamanian frogs. Front Microbiol 9: 298. 29520260 10.3389/fmicb.2018.00298 PMC5826957

[ref104] Vitousek PM , Mooney HA, Lubchenco J, Melillo JM (1997) Human domination of Earth’s ecosystems. Science 277: 494–499.

[ref105] Warne RW , Crespi EJ, Brunner JL (2011) Escape from the pond: stress and developmental responses to ranavirus infection in wood frog tadpoles. Funct Ecol 25: 139–146.

[ref106] Werner EE , Glennemeier KS (1999) Influence of forest canopy cover on the breeding pond distributions of several amphibian species. Copeia 1999: 1–12.

[ref107] Wickham H (2016) ggplot2: Elegant Graphics for Data Analysis. R Package Version 3.1.1. Springer-Verlag, New York, NY, USA. ISBN 978-3-319-24277-4. https://ggplot2.tidyverse.org

[ref108] Wikelski M , Cooke SJ (2006) Conservation physiology. Trends Ecol Evol 21: 38–46. 16701468 10.1016/j.tree.2005.10.018

[ref109] Wingfield JC , Romero LM (2001) Adrenocortical responses to stress and their modulation in free-living vertebrates. In BS McEwen, ed, Handbook of Physiology, Section 7: the Endocrine System. Oxford University Press, New York, NY, USA, pp. 211–234.

[ref110] Woodhams DC , Ardipradja K, Alford RA, Marantelli G, Reinert LK, Rollins-Smith LA (2007) Resistance to chytridiomycosis varies among amphibian species and is correlated with skin peptide defenses. Anim Conserv 10: 409–417.

[ref111] Woodhams DC , Bletz M, Keuneman J, McKenzie V (2016) Managing amphibian disease with skin microbiota. Trends Microbiol 24: 161–164. 26916805 10.1016/j.tim.2015.12.010

[ref112] Woodhams DC , Brandt H, Baumgartner S, Kielgast J, Kupfer E, Tobler U, Davis LR, Schmidt BR, Bel C, Hodel S et al. (2014) Interacting symbionts and immunity in the amphibian skin mucosome predict disease risk and probiotic effectiveness. PLoS One 9: e96375. 24789229 10.1371/journal.pone.0096375 PMC4005770

