# Supplementary material for: Changes in physiology and microbial diversity in larval ornate chorus frogs are associated with habitat quality
Source: Conserv Physiol. 2020 Jun 15;8(1):coaa047. doi: 10.1093/conphys/coaa047 (PMC7294888; doi:10.1093/conphys/coaa047)
Supplement: Supplementary_Material_coaa047 [file supplementary_material_coaa047.doc]

**Supplementary Material**

**Table S1:** Sample sizes for water-borne CORT (baseline and agitation treatments), mucosome function, and skin bacterial diversity measures collected from each Site across both sampling years. Mucosome function and bacterial diversity were only measured in 2017. AP = Apalachicola National Forest, EG = Eglin Air Force Base, JC = Joseph W. Jones Ecological Research Center at Ichauway, LF = Lafayette Forest Wildlife Environmental Area, SM = St. Marks National Wildlife Refuge, OS = Orianne Society Preserve, WC = James W. Webb Wildlife Center.

| Sample Sizes | | | | | | |
| --- | --- | --- | --- | --- | --- | --- |
| Year | Site | Total | Baseline CORT | Agitation CORT | Mucosome | Diversity |
| 2016 | EG1 | 34 | 17 | 17 | NA | NA |
|  | AP1 | 37 | 18 | 19 | NA | NA |
|  | AP2 | 28 | 14 | 14 | NA | NA |
|  | AP3 | 35 | 17 | 18 | NA | NA |
|  | SM1 | 37 | 18 | 19 | NA | NA |
|  | SM2 | 28 | 14 | 14 | NA | NA |
|  | JC1 | 38 | 18 | 20 | NA | NA |
|  | JC2 | 38 | 18 | 20 | NA | NA |
|  | JC3 | 38 | 18 | 20 | NA | NA |
|  | LF1 | 30 | 15 | 15 | NA | NA |
|  | *Total* | *343* | *167* | *176* | *NA* | *NA* |
| 2017 | EG1 | 39 | 19 | 20 | 10 | 10 |
|  | EG2 | 30 | 15 | 15 | 10 | 11 |
|  | AP1 | 35 | 15 | 20 | 10 | 10 |
|  | AP2 | 30 | 15 | 15 | 10 | 11 |
|  | SM3 | 40 | 20 | 20 | 10 | NA |
|  | SM4 | 30 | 15 | 15 | 10 | 9 |
|  | JC1 | 30 | 15 | 15 | 9 | 10 |
|  | JC2 | 39 | 20 | 19 | 10 | 9 |
|  | JC3 | 39 | 20 | 19 | 9 | 9 |
|  | OS1 | 30 | 15 | 15 | 10 | 10 |
|  | OS2 | 40 | 20 | 20 | 10 | NA |
|  | WC1 | 40 | 20 | 20 | 9 | 9 |
|  | *Total* | *422* | *209* | *213* | *117* | *98* |

**Table S2:** Baseline and agitation CORT release rates of *Pseudacris ornata* tadpoles from the 10 ponds sampled in 2016 and 12 ponds sampled in 2017 were modeled as a function of the following predictors.

| Model number | Predictors |
| --- | --- |
| 1 | Landcover100 |
| 2 | Urban1000 |
| 3 | Canopy500 |
| 4 | PC1 |
| 5 | PC2 |
| 6 | PC1+PC2 |
| 7 | Landcover100+PC1 |
| 8 | Landcover100+PC2 |
| 9 | Landcover100+PC1+PC2 |
| 10 | Canopy500+PC1 |
| 11 | Canopy500+PC2 |
| 12 | Canopy500+PC1+PC2 |
| 13 | Urban1000+PC1 |
| 14 | Urban1000+PC2 |
| 15 | Urban1000+PC1+PC2 |
| 16 | MEM2 |
| 17 | MEM3 |
| 18 | MEM2+MEM3 |
| 19 | MEM2+PC1 |
| 20 | MEM3+PC1 |
| 21 | MEM2+MEM3+PC1 |
| 22 | MEM2+PC2 |
| 23 | MEM3+PC2 |
| 24 | MEM2+MEM3+PC2 |
| 25 | MEM2+PC1+PC2 |
| 26 | MEM3+PC1+PC2 |
| 28  29 | MEM2+MEM3+PC1+PC2  Property |

**Table S3:** Mucosome function of *Pseudacris ornata* tadpoles sampled in 2017 were modeled as a function of the following predictors.

| Model number | Predictors |
| --- | --- |
| 1 | Landcover1000 |
| 2 | Urban100 |
| 3 | Canopy500 |
| 4 | PC1 |
| 5 | PC2 |
| 6 | PC1+PC2 |
| 7 | Landcover1000+PC1 |
| 8 | Landcover1000+PC2 |
| 9 | Landcover1000+PC1+PC2 |
| 10 | Canopy500+PC1 |
| 11 | Canopy500+PC2 |
| 12 | Canopy500+PC1+PC2 |
| 13 | Urban100+PC1 |
| 14 | Urban100+PC2 |
| 15 | Urban100+PC1+PC2 |
| 16 | MEM2 |
| 17 | MEM3 |
| 18 | MEM2+MEM3 |
| 19 | MEM2+PC1 |
| 20 | MEM3+PC1 |
| 21 | MEM2+MEM3+PC1 |
| 22 | MEM2+PC2 |
| 23 | MEM3+PC2 |
| 24 | MEM2+MEM3+PC2 |
| 25 | MEM2+PC1+PC2 |
| 26 | MEM3+PC1+PC2 |
| 27 | MEM2+MEM3+PC1+PC2 |
| 28  29 | BCORT  Property |

**Table S4:** Skin bacterial diversity (Richness, Shannon, Simpson) of *Pseudacris ornata* tadpoles sampled in 2017 were modeled as a function of the following predictors.

| Model number | Predictors |
| --- | --- |
| 1 | Landcover1000 |
| 2 | Urban500 |
| 3 | Canopy100 |
| 4 | PC1 |
| 5 | PC2 |
| 6 | PC1+PC2 |
| 7 | Landcover1000+PC1 |
| 8 | Landcover1000+PC2 |
| 9 | Landcover1000+PC1+PC2 |
| 10 | Canopy100+PC1 |
| 11 | Canopy100+PC2 |
| 12 | Canopy100+PC1+PC2 |
| 13 | Urban500+PC1 |
| 14 | Urban500+PC2 |
| 15 | Urban500+PC1+PC2 |
| 16 | MEM2 |
| 17 | MEM3 |
| 18 | MEM2+MEM3 |
| 19 | MEM2+PC1 |
| 20 | MEM3+PC2 |
| 21 | MEM2+PC1+PC2 |
| 22 | MEM3+PC1+PC2 |
| 23 | MEM3+PC2 |
| 24 | MEM3+PC1+PC2 |
| 25 | MEM2+MEM3+PC1 |
| 26 | MEM2+MEM3+PC2 |
| 27 | MEM2+MEM3+PC1+PC2 |
| 28 | BCORT |

**Table S5: Top models explaining environmental influences on *Psuedacris ornata* natural log-transformed corticosterone release rates (CORT) from 10 sites sampled in 2016. Models are ranked according to Akaike's Information Criterion adjusted for small sample size (AICc). AICc score, change in AICc (ΔAICc), and AICc model weight (ω) for each model are shown for the top ranked models (ΔAICc < 2).**

| Response | Model | K | AICc | ΔAICc | ω |
| --- | --- | --- | --- | --- | --- |
| Baseline CORT | MEM2+PC1+PC2 | 6 | 315.76 | 0.00 | 0.150 |
|  | PC1+PC2 | 5 | 316.60 | 0.84 | 0.098 |
|  | MEM2+MEM3+PC1+PC2 | 7 | 317.20 | 1.44 | 0.073 |
|  | PC1 | 4 | 317.28 | 1.52 | 0.070 |
|  | MEM2+PC1 | 5 | 317.33 | 1.58 | 0.068 |
|  | MEM3+PC2 | 5 | 317.65 | 1.89 | 0.058 |
| Agitation CORT | MEM3+PC2 | 5 | 303.25 | 0.00 | 0.166 |
|  | PC1+PC2 | 5 | 303.45 | 0.20 | 0.150 |
|  | Canopy500+PC1+PC2 | 6 | 303.90 | 0.65 | 0.120 |
|  | MEM2+PC1+PC2 | 6 | 304.18 | 0.93 | 0.104 |
|  | MEM2+MEM3+PC2 | 6 | 304.87 | 1.62 | 0.074 |

**Table S6: Top models explaining environmental influences on *Psuedacris ornata* natural log-transformed corticosterone release rates (CORT), mucosome function, and bacterial diversity (Richness, Shannon, Simpson) from 12 sites sampled in 2017. Models are ranked according to Akaike's Information Criterion adjusted for small sample size (AICc). AICc score, change in AICc (ΔAICc), and AICc model weight (ω) for each model are shown for the top ranked models (ΔAICc < 2).**

| Response | Model | K | AICc | ΔAICc | ω |
| --- | --- | --- | --- | --- | --- |
| Baseline CORT | Urban1000 | 4 | 342.71 | 0.00 | 0.388 |
|  | Urban1000+PC2 | 5 | 344.18 | 1.48 | 0.185 |
| Agitation CORT | Urban1000 | 4 | 316.85 | 0.00 | 0.226 |
|  | Urban1000+PC1 | 5 | 318.75 | 1.90 | 0.088 |
| Mucosome function | PC2 | 4 | 950.14 | 0.00 | 0.145 |
|  | Urban100 | 4 | 951.13 | 0.99 | 0.088 |
|  | Urban100+PC2 | 5 | 951.34 | 1.20 | 0.080 |
|  | Canopy500+PC2 | 5 | 951.77 | 1.62 | 0.064 |
|  | PC1+PC2 | 5 | 952.06 | 1.92 | 0.056 |
| Richness | Landcover1000 | 5 | 1289.06 | 0.00 | 0.314 |
|  | Landcover1000+PC1 | 6 | 1290.78 | 1.72 | 0.133 |
| Shannon diversity | MEM2 | 4 | 270.95 | 0.00 | 0.124 |
|  | Canopy100+PC2 | 5 | 271.49 | 0.54 | 0.094 |
|  | Urban500+PC1 | 5 | 271.85 | 0.90 | 0.079 |
|  | Canopy100 | 4 | 271.90 | 0.95 | 0.077 |
|  | Urban500 | 4 | 272.58 | 1.63 | 0.055 |
|  | MEM2+PC1 | 5 | 272.65 | 1.70 | 0.053 |
| Simpson diversity | MEM2 | 4 | -96.70 | 0.00 | 0.312 |
|  | MEM2+MEM3 | 5 | -95.10 | 1.60 | 0.140 |

**Table S7:** Results of Welch t-tests comparing agitation and baseline corticosterone release rates for each site across both sampling years. Values in bold indicate significant differences (*p* < 0.05). AP = Apalachicola National Forest, EG = Eglin Air Force Base, JC = Joseph W. Jones Ecological Research Center at Ichauway, LF = Lafayette Forest Wildlife Environmental Area, SM = St. Marks National Wildlife Refuge, OS = Orianne Society Preserve, WC = James W. Webb Wildlife Center.

| Year | Site | df | t | *P* |
| --- | --- | --- | --- | --- |
| 2016 | EG1 | 31.6 | 2.19 | **0.018** |
|  | AP1 | 32.4 | 1.75 | **0.044** |
|  | AP2 | 16.1 | 1.98 | **0.032** |
|  | AP3 | 32.9 | 1.22 | 0.115 |
|  | SM1 | 32.5 | 0.05 | 0.481 |
|  | SM2 | 20.3 | 0.52 | 0.304 |
|  | JC1 | 26.2 | 2.06 | **0.024** |
|  | JC2 | 35.4 | 2.01 | **0.026** |
|  | JC3 | 35.8 | 0.08 | 0.467 |
|  | LF1 | 19.8 | 2.43 | **0.012** |
| 2017 | EG1 | 35.7 | 2.41 | **0.011** |
|  | EG2 | 27.9 | 0.08 | 0.207 |
|  | AP1 | 24.8 | -1.16 | 0.871 |
|  | AP2 | 25.6 | 1.55 | 0.067 |
|  | SM3 | 27.0 | 2.87 | **0.004** |
|  | SM4 | 27.9 | 1.78 | **0.043** |
|  | JC1 | 25.6 | -0.62 | 0.729 |
|  | JC2 | 36.9 | 1.81 | **0.039** |
|  | JC3 | 35.6 | -0.58 | 0.718 |
|  | OS1 | 24.8 | 3.17 | **0.002** |
|  | OS2 | 33.3 | 1.48 | 0.074 |
|  | WC1 | 37.7 | 4.15 | **<0.0001** |

**Table S8:** Highest and lowest mean ± SE values for each of three alpha diversity indices (Richness, Shannon, and Simpson) for both Sites and Properties sampled in 2017. AP = Apalachicola National Forest, EG = Eglin Air Force Base, JC = Joseph W. Jones Ecological Research Center at Ichauway, WC = James W. Webb Wildlife Center.

| Diversity index | Site | Mean ± SE |  | Property | Mean ± SE |
| --- | --- | --- | --- | --- | --- |
| Richness |  |  |  |  |  |
| Highest | JC2 | 562.56 ± 48.23 |  | WC | 467.44 ± 64.67 |
| Lowest | AP2 | 121.82 ± 10.08 |  | AP | 171.00 ± 23.10 |
| Shannon |  |  |  |  |  |
| Highest | JC3 | 3.46 ± 0.33 |  | JC | 3.41 ± 0.16 |
| Lowest | EG2 | 2.21 ± 0.31 |  | EG | 2.53 ± 0.25 |
| Simpson (evenness) |  |  |  |  |  |
| Highest | JC3 | 0.89 ± 0.02 |  | JC | 0.87 ± 0.01 |
| Lowest | EG2 | 0.65 ± 0.07 |  | EG | 0.73 ± 0.05 |

**Table S9:** Results of *post-hoc* pairwise comparisons among ponds to examine the similarity of OTU beta diversity from *Pseudacris ornata* skin microbial communities. AP = Apalachicola National Forest, EG = Eglin Air Force Base, JC = Joseph W. Jones Ecological Research Center at Ichauway, LF = Lafayette Forest Wildlife Environmental Area, SM = St. Marks National Wildlife Refuge, OS = Orianne Society Preserve, WC = James W. Webb Wildlife Center.

| Comparison | F | *P* |
| --- | --- | --- |
| AP1 x AP2 | 1.46 | 0.144 |
| AP1 x EG1 | 3.68 | 0.001 |
| AP1 x EG2 | 7.73 | 0.001 |
| AP1 x JC1 | 6.48 | 0.001 |
| AP1 x JC2 | 9.11 | 0.001 |
| AP1 x JC3 | 7.78 | 0.001 |
| AP1 x OS1 | 6.66 | 0.001 |
| AP1 x SM4 | 1.58 | 0.117 |
| AP1 x WC1 | 6.46 | 0.001 |
| AP2 x EG1 | 4.06 | 0.001 |
| AP2 x EG2 | 9.75 | 0.001 |
| AP2 x JC1 | 7.08 | 0.001 |
| AP2 x JC2 | 9.87 | 0.001 |
| AP2 x JC3 | 8.34 | 0.001 |
| AP2 x OS1 | 8.38 | 0.001 |
| AP2 x SM4 | 2.31 | 0.033 |
| AP2 x WC1 | 7.75 | 0.001 |
| EG1 x EG2 | 9.04 | 0.001 |
| EG1 x JC1 | 4.91 | 0.001 |
| EG1 x JC2 | 6.34 | 0.001 |
| EG1 x JC3 | 6.63 | 0.001 |
| EG1 X OS1 | 6.89 | 0.001 |
| EG1 X SM4 | 4.96 | 0.001 |
| EG1 X WC1 | 6.86 | 0.001 |
| EG2 X JC1 | 15.82 | 0.001 |
| EG2 X JC2 | 21.63 | 0.001 |
| EG2 X JC3 | 21.15 | 0.001 |
| EG2 X OS1 | 21.95 | 0.001 |
| EG2 X SM4 | 13.32 | 0.001 |
| EG2 X WC1 | 18.54 | 0.001 |
| JC1 X JC2 | 6.52 | 0.001 |
| JC1 X JC3 | 4.46 | 0.001 |
| JC1 X OS1 | 6.89 | 0.001 |
| JC1 X SM4 | 10.48 | 0.001 |
| JC1 X WC1 | 9.94 | 0.001 |
| JC2 X JC3 | 12.74 | 0.001 |
| JC2 X OS1 | 11.49 | 0.001 |
| JC2 X SM4 | 14.47 | 0.001 |
| JC2 X WC1 | 15.54 | 0.001 |
| JC3 X OS1 | 8.23 | 0.001 |
| JC3 X SM4 | 13.68 | 0.001 |
| JC3 X WC1 | 13.09 | 0.001 |
| OS1 X SM4 | 11.44 | 0.001 |
| OS1 X WC1 | 13.7 | 0.001 |
| SM4 X WC1 | 10.04 | 0.001 |

**Table S10:** Results of *post-hoc* pairwise comparisons among Properties to examine the similarity of OTU beta diversity from *Pseudacris ornata* skin microbial communities. AP = Apalachicola National Forest, EG = Eglin Air Force Base, JC = Joseph W. Jones Ecological Research Center at Ichauway, LF = Lafayette Forest Wildlife Environmental Area, SM = St. Marks National Wildlife Refuge, OS = Orianne Society Preserve, WC = James W. Webb Wildlife Center.

| Comparison | F | *P* |
| --- | --- | --- |
| AP X EG | 6.09 | 0.001 |
| AP X JC | 11.74 | 0.001 |
| AP X OS | 8.35 | 0.001 |
| AP X SM | 1.80 | 0.064 |
| AP X WC | 7.82 | 0.001 |
| EG X JC | 10.58 | 0.001 |
| EG X OS | 9.93 | 0.001 |
| EG X SM | 5.65 | 0.001 |
| EG X WC | 8.60 | 0.001 |
| JC X OS | 6.46 | 0.001 |
| JC X SM | 10.85 | 0.001 |
| JC X WC | 10.60 | 0.001 |
| OS X SM | 11.44 | 0.001 |
| OS X WC | 13.70 | 0.001 |
| SM X WC | 10.04 | 0.001 |

**
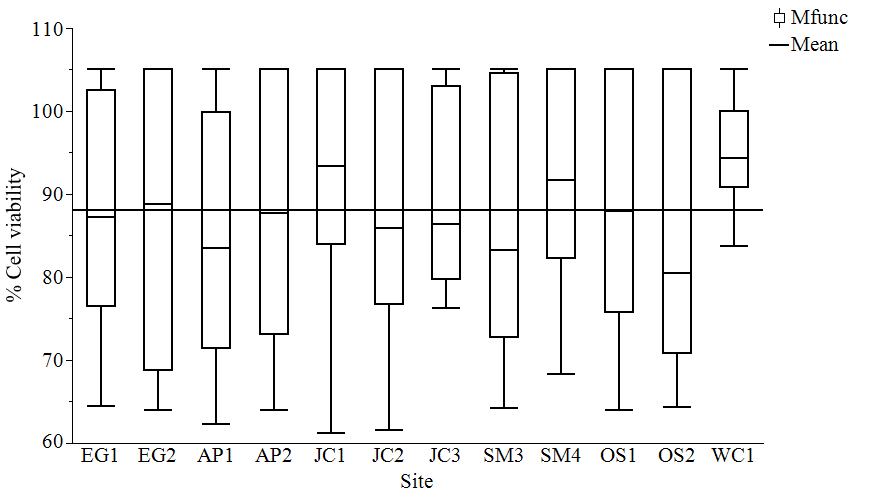
**

**Figure S1:** Box and whisker plots showing percent cell viability of *Batrachochytrium dendrobatidis* when plated along with a sample of the water containing the mucosome of 9 *Pseudacris ornata* tadpoles from each pond, representing the mucosome function. Box and whisker plots indicate median, interquartiles, and range for each pond. Horizontal line denotes average percent cell viability. Mucosome samples were only collected from ponds sampled in 2017. Ponds: AP = Apalachicola National Forest, EG = Eglin Air Force Base, JC = Joseph W. Jones Ecological Research Center at Ichauway, LF = Lafayette Forest Wildlife Environmental Area, SM = St. Marks National Wildlife Refuge, OS = Orianne Society Preserve, WC = James W. Webb Wildlife Center. Ponds are ordered geographically from west to east.
